# Multimodal analysis of cell-free DNA whole-genome sequencing for pediatric cancers with low mutational burden

**DOI:** 10.1038/s41467-021-23445-w

**Published:** 2021-05-28

**Authors:** Peter Peneder, Adrian M. Stütz, Didier Surdez, Manuela Krumbholz, Sabine Semper, Mathieu Chicard, Nathan C. Sheffield, Gaelle Pierron, Eve Lapouble, Marcus Tötzl, Bekir Ergüner, Daniele Barreca, André F. Rendeiro, Abbas Agaimy, Heidrun Boztug, Gernot Engstler, Michael Dworzak, Marie Bernkopf, Sabine Taschner-Mandl, Inge M. Ambros, Ola Myklebost, Perrine Marec-Bérard, Susan Ann Burchill, Bernadette Brennan, Sandra J. Strauss, Jeremy Whelan, Gudrun Schleiermacher, Christiane Schaefer, Uta Dirksen, Caroline Hutter, Kjetil Boye, Peter F. Ambros, Olivier Delattre, Markus Metzler, Christoph Bock, Eleni M. Tomazou

**Affiliations:** 1 St. Anna Children’s Cancer Research Institute (CCRI), Vienna, Austria; 2 INSERM U830, Équipe Labellisée LNCC, PSL Research University, SIREDO Oncology Centre, Institut Curie Research Centre, Paris, France; 3 Balgrist University Hospital, University of Zurich, Zurich, Switzerland; 4 Department of Pediatrics, University Hospital Erlangen, Erlangen, Germany; 5 Center for Public Health Genomics, University of Virginia, Charlottesville, VA USA; 6 Unité de Génétique Somatique, Service d’oncogénétique, Institut Curie, Centre Hospitalier, Paris, France; 7 CeMM Research Center for Molecular Medicine of the Austrian Academy of Sciences, Vienna, Austria; 8 Institute of Pathology, University Hospital Erlangen, Erlangen, Germany; 9 St. Anna Kinderspital, Department of Pediatrics, Medical University, Vienna, Austria; 10 Department of Tumor Biology, Institute for Cancer Research, Oslo University Hospital, Oslo, Norway; 11 Department of Clinical Science, University of Bergen, Bergen, Norway; 12 Pediatric Department, Hematology and Oncology Pediatric Institute, Centre Léon Bérard, Lyon, France; 13 Children’s Cancer Research Group, Leeds Institute of Medical Research, St. James’s University Hospital, Leeds, UK; 14 Department of Pediatric Oncology, Royal Manchester Children’s Hospital, Manchester, UK; 15 Department of Oncology, UCL Cancer Institute, London, UK; 16 Department of Oncology, University College London Hospital, London, UK; 17 University Hospital Essen, Pediatrics III, West German Cancer Centre, Essen, Germany; 18 Department of Oncology, Oslo University Hospital, The Norwegian Radium Hospital, Oslo, Norway; 19 Institute of Artificial Intelligence, Center for Medical Statistics, Informatics, and Intelligent Systems, Medical University of Vienna, Vienna, Austria; 20 Ludwig Boltzmann Institute for Rare and Undiagnosed Diseases, Vienna, Austria

**Keywords:** Cancer genomics, Software, Epigenomics, Biomarkers, Paediatric cancer

## Abstract

Sequencing of cell-free DNA in the blood of cancer patients (liquid biopsy) provides attractive opportunities for early diagnosis, assessment of treatment response, and minimally invasive disease monitoring. To unlock liquid biopsy analysis for pediatric tumors with few genetic aberrations, we introduce an integrated genetic/epigenetic analysis method and demonstrate its utility on 241 deep whole-genome sequencing profiles of 95 patients with Ewing sarcoma and 31 patients with other pediatric sarcomas. Our method achieves sensitive detection and classification of circulating tumor DNA in peripheral blood independent of any genetic alterations. Moreover, we benchmark different metrics for cell-free DNA fragmentation analysis, and we introduce the LIQUORICE algorithm for detecting circulating tumor DNA based on cancer-specific chromatin signatures. Finally, we combine several fragmentation-based metrics into an integrated machine learning classifier for liquid biopsy analysis that exploits widespread epigenetic deregulation and is tailored to cancers with low mutation rates. Clinical associations highlight the potential value of cfDNA fragmentation patterns as prognostic biomarkers in Ewing sarcoma. In summary, our study provides a comprehensive analysis of circulating tumor DNA beyond recurrent genetic aberrations, and it renders the benefits of liquid biopsy more readily accessible for childhood cancers.

## Abstract (web summary)

Liquid biopsies enable minimally invasive applications for diagnosis and treatment monitoring. Here the authors analyse fragmentation patterns of circulating tumour DNA on multiple levels and develop a bioinformatic tool, LIQUORICE, to accurately detect and classify paediatric cancers with low mutational burden.

## Introduction

Liquid biopsy analysis of circulating cell-free DNA (cfDNA) from peripheral blood has emerged as a valuable diagnostic tool in oncology^1–5^. Sample collection is quick and minimally invasive, thus allowing longitudinal analysis with high temporal resolution. In cancer patients, cfDNA consists in part of cancer-derived circulating tumor DNA (ctDNA), and it has been shown that tumor-related genetic and epigenetic alterations can be detected by analyzing cfDNA in cancer patients^6–14^. As a consequence, cfDNA analysis holds great promise for precision oncology and personalized therapies, and is currently evaluated in a broad range of clinical studies^15,16^.

In pediatric tumors, high levels of tumor-derived DNA in blood have been linked to poor clinical outcome^17^, and initial studies illustrate the value of liquid biopsy analysis for disease monitoring^18,19^. The analysis of cfDNA in childhood cancers has focused primarily on tumor-specific genetic aberrations, including chromosomal translocations (fusion genes) and copy-number alternations (CNAs), using assays such as droplet digital PCR (ddPCR), targeted NGS panels, exome sequencing, and low-coverage whole-genome sequencing^17,19–27^. These approaches depend on the presence of readily detectable genetic aberrations, require prior knowledge of chromosomal breakpoints (ddPCR), focus on only one or a few genetic alterations (ddPCR, targeted NGS panels), and may suffer from low sensitivity^6,28,29^. There is thus an unmet need for new approaches to liquid biopsy analysis in pediatric tumors, particularly accounting for the low rate of recurrent genetic alterations observed in most childhood cancers^30,31^.

**Fig. 1:**
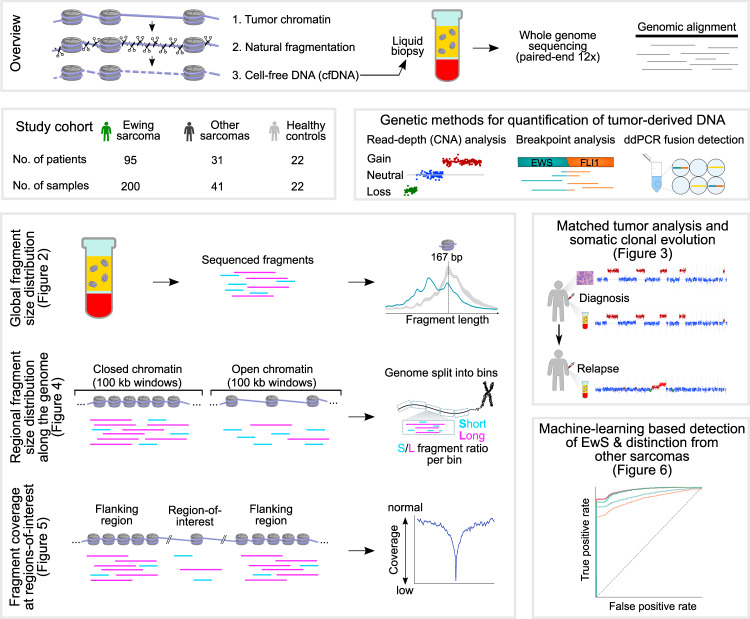
Whole-genome sequencing of cfDNA enables fragment-based liquid biopsy analysis in Ewing sarcoma. The top row of the figure describes the fragmentation and fragment-based analysis of cfDNA in cancer patients. The center row introduces the study cohort (center left) and illustrates the quantification of tumor-derived DNA based on genetic evidence, which is used as a reference in this study (center right). The bottom part of the figure outlines three complementary approaches to fragment-based cfDNA analyses: global fragment-size distribution; regional fragment-size distribution along the genome; and fragment coverage at regions-of-interest (bottom left). CNA profiles were used for comparing cfDNA to matched tumors biopsies and for time-resolved monitoring of tumor evolution. Fragment-based cfDNA metrics were combined for machine learning-based tumor detection and classification (bottom right). The main figures describing each of the analyses are indicated in brackets.

Recent studies indicate that cfDNA fragmentation patterns provide complementary information to the genetic analysis of somatic mutations and copy-number aberrations. Given the low rate of genetic alterations in many childhood cancers, such fragmentation-based methods could be of high relevance for pediatric oncology. These methods are based on the intriguing observation that the fragmentation of DNA from dying tumor cells is neither random nor determined solely by DNA sequence; rather, it appears to reflect the chromatin structure and epigenetic states of the cells from which the DNA fragments were derived^32–35^ (Fig. 1). Given that many pediatric tumors harbor highly characteristic epigenetic aberrations^36–44^, analyzing cfDNA fragmentation patterns may not only help quantify ctDNA in the absence of recurrent genetic aberrations, but it may also provide minimally invasive insights into the tumor’s epigenetic state at diagnosis, relapse, and over the course of therapy.

Ewing sarcoma (EwS)^45^ constitutes an ideal model cancer for establishing and validating fragment-based liquid biopsy analysis for pediatric tumors. EwS has a unique epigenetic signature with established clinical associations^41^, which constitutes a potential epigenetic marker for early diagnosis and tumor classification based on cfDNA. Moreover, EwS tumors have well-established genetic aberrations that can be used for comparison, most notably the tumor-defining chromosomal translocation between *EWSR1* and an *Ets* family member gene (most commonly *FLI1*), and a small number of recurrent CNAs^46–48^.

In this work, we establish a reference data set of cfDNA whole-genome sequencing profiles for a large collection of patients with EwS and other pediatric sarcomas. We present an integrative analysis and comparison of fragmentation patterns in this data set (Fig. 1), including (i) global fragment-size distribution; (ii) regional fragment-size distribution along the genome; and (iii) fragment coverage at predefined regions-of-interest. We show that tumor DNA in the blood of patients with EwS is highly and characteristically fragmented, we identify an EwS-specific epigenetic signature among regional fragmentation patterns across the genome, and we introduce a bioinformatic method for accurate quantification of these epigenetic signatures in cfDNA. Moreover, we investigate the clinical associations of cfDNA fragmentation patterns, and we introduce a machine learning method that integrates multiple cfDNA fragment-based metrics into highly predictive models for the detection and classification of pediatric solid tumors. In summary, we present one of the largest cfDNA sequencing studies for childhood cancer, resulting in a detailed genetic and epigenetic analysis of EwS tumors using liquid biopsies. Our study contributes to liquid biopsy methodology by introducing an integrated, broadly applicable, method for detection and quantification of epigenetic signatures based on cfDNA fragmentation patterns.

## Results

### Deep whole-genome sequencing of cell-free DNA uncovers tumor-specific fragmentation patterns

To establish a comprehensive data set for liquid biopsy analysis in pediatric sarcomas, we performed whole-genome sequencing with a median coverage of 12× for 263 cfDNA samples collected from EwS patients (*n* = 95), other pediatric sarcomas (*n* = 31), and healthy controls (*n* = 22) (Fig. 1 and Supplementary Data 1). Where possible, we included samples from the same patient at multiple stages of cancer (at diagnosis and during therapy, remission, and relapse), to be able to monitor disease courses in individual patients.

We first performed genetic analysis of the cfDNA samples based on the whole-genome sequencing data and, independently, based on ddPCR experiments targeting the EwS-specific *EWS-Ets* fusion oncogene. This genetic analysis allowed us to estimate the percentage of tumor-derived DNA for each cfDNA sample, and it provided a reference for the fragment-based analysis (Fig. 1). We applied three methods for the genetic analysis of cfDNA in pediatric sarcomas: (i) CNA quantification based on read depth using ichorCNA^7^; (ii) quantification of the *EWS-Ets* fusion oncogene from the whole-genome sequencing data^17^; and (iii) *EWS-Ets* quantification using ddPCR^23^. Based on the combination of these genetic methods, we detected tumor-derived DNA in 99 cfDNA samples (from 61 patients with EwS), of which 59 had more than 20% tumor content in cfDNA (Supplementary Data 2).

**Fig. 2:**
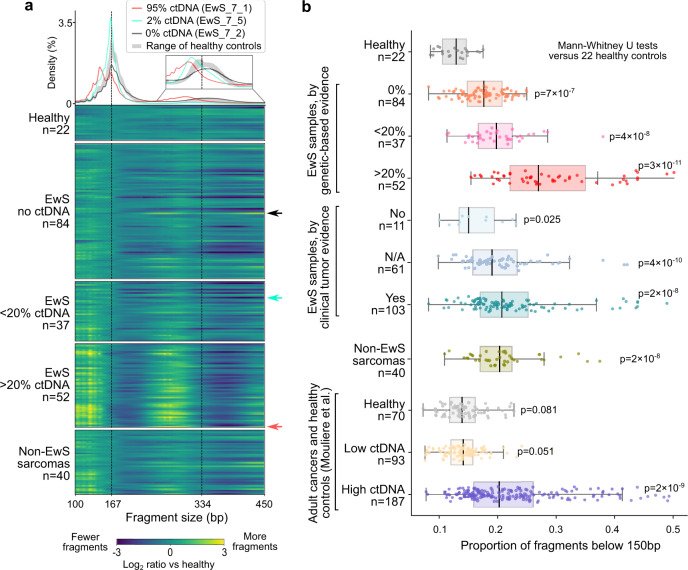
Global fragment-size analysis detects highly fragmented EwS tumor DNA. **a** Histogram (top) showing the cfDNA fragment-size distribution for three representative samples with high (95%), low (2%), and undetectable (0%) tumor-derived DNA (ctDNA) content. The range of cfDNA fragment sizes in 22 healthy controls is shown in gray. Heatmap (bottom) showing the relative fragment-size distribution of 235 cfDNA samples subjected to whole-genome sequencing, each normalized against the median of 22 healthy controls. EwS samples are grouped by genetically inferred tumor-derived DNA content. The three samples shown in the histogram are marked by arrows. **b** Proportion of short cfDNA fragments (20-150 bp) for pediatric sarcomas and healthy controls (data from this study) and for adult cancers (published data^49^). Boxes correspond to interquartile ranges (IQR), black lines indicate the median, and the whiskers extend to the lowest or highest data points that are still within 1.5 IQR of the bottom or top quartile, respectively. Significance versus the 22 healthy controls was assessed using two-sided Mann–Whitney *U* tests.

Next, we analyzed global DNA fragmentation patterns as a non-genetic way of detecting tumor-derived DNA in the cfDNA samples (Fig. 2a), building upon recent reports suggesting that DNA fragmentation patterns of cfDNA reflect the chromatin profiles of the cells from which the cfDNA is derived^32–35^. We observed a global shift toward shorter fragments in the cfDNA of patients with EwS compared to healthy controls, especially for the characteristic 167 bp peak of cfDNA, which corresponds to the length of DNA bound by one nucleosome plus linker DNA.

We quantified these tumor-induced changes using published cfDNA metrics^49^, most notably the proportion of short fragments with sizes below 150 bp (Supplementary Data 3). This proportion of short fragments was consistently higher in cfDNA samples from patients with EwS than from healthy controls (*p* < 0.001, Mann–Whitney *U* test; Fig. 2b). The trend toward shorter fragment sizes was similarly pronounced as in patients with adult cancers known for their high levels of tumor-derived DNA and high fragmentation in cfDNA, such as lung and colorectal cancers^49^ (Fig. 2b). We also observed similar fragmentation patterns for cfDNA samples from patients with other pediatric sarcomas (Supplementary Fig. 1a). Our analysis of cfDNA fragmentation patterns strongly suggests that pediatric sarcoma-derived DNA is more fragmented than cfDNA from other sources (e.g., from dying blood and tissue cells). This conclusion was further supported by the fact that cfDNA samples with high tumor-derived DNA content (based on genetic evidence) had the highest proportion of short fragments (Fig. 2b).

Interestingly, many cfDNA samples from patients that did not show genetic evidence of tumor-derived DNA still had a higher proportion of short fragments compared to those obtained from healthy controls (Fig. 2b and Supplementary Fig. 1a). We thus hypothesized that our analysis of the global fragment-size distribution detects low levels of tumor-derived DNA not seen using genetic markers. Indeed, for five patients with EwS we observed a high proportion of short reads and high tumor content in cfDNA based on *EWS-Ets* quantification, but no detectable CNAs (Supplementary Fig. 1b and Supplementary Data 2). Such copy-number neutral cases, which are common in pediatric tumors^30^, highlight the potential clinical value of non-genetic methods for analyzing cfDNA based on fragmentation patterns.

In summary, we observed a characteristic global fragment-size distribution in cfDNA from patients with pediatric sarcomas, similar to previous reports for adult cancers^49,50^. These fragmentation patterns may be exploited for detection and quantification of tumor-derived DNA independent of any genetic aberrations, which is particularly relevant for pediatric tumors with few genetic lesions.

### Fragment-size filtering of cfDNA profiles enhances CNA detection and improves monitoring of clonal evolution

We can exploit the observation that short cfDNA fragments tend to be tumor-derived, in order to enrich for these DNA fragments in the genetic analysis of CNAs^49,50^ and thereby refine the mapping of tumor-associated CNAs^46–48^. In EwS, CNAs are more frequent than recurrent somatic mutations^30^, and they are being investigated as potential biomarkers^51^. Moreover, accurate CNA profiles can support the analysis of clonal heterogeneity and evolutionary history^52^. We thus evaluated whether filtering for short fragments enhances CNA detection in our data set.

**Fig. 3:**
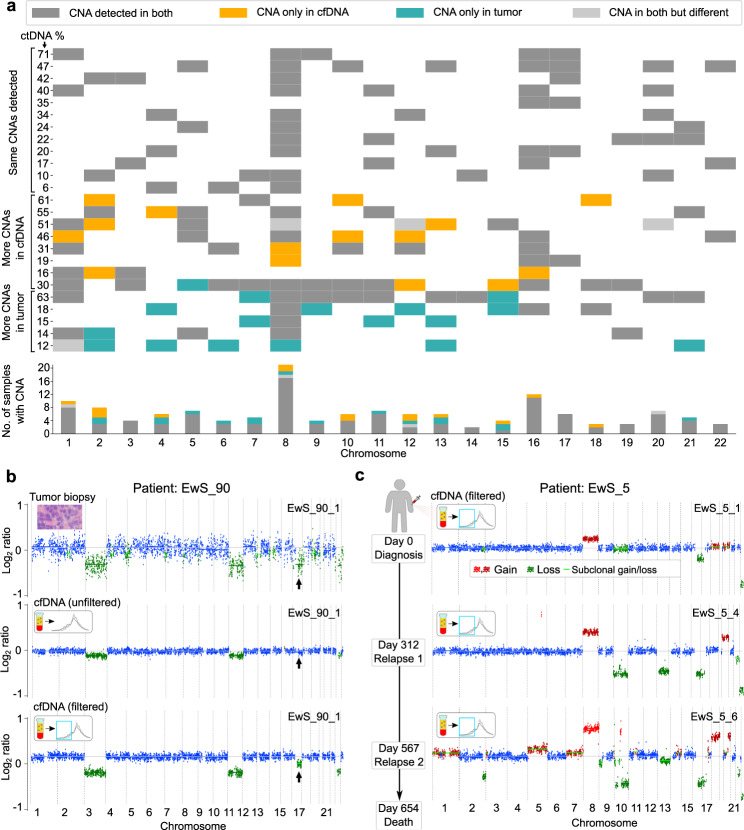
CNA profiles in liquid biopsies reflect tumor aberrations and allow monitoring of tumor evolution. **a** Comparison of CNAs detected in cfDNA versus matched tumor biopsies. Only sample pairs with tumor-derived DNA detected in cfDNA based on ichorCNA are shown (*n* = 29 sample pairs); four copy-number neutral sample pairs were omitted from the plot. Patients are grouped according to CNA state in cfDNA relative to the matched tumor biopsy. Gray represents CNAs detected in both cfDNA and matched tumor biopsy, orange indicates CNAs detected only in cfDNA, and turquoise represents CNAs detected only in the tumor biopsy. The CNAs detected in cfDNAs versus matched tumor biopsies are summarized in a bar plot (bottom). **b** CNA plot (ichorCNA) of an EwS cfDNA sample (EwS_90_1) before (middle) and after (bottom) in silico size selection to the range of 90–150 bp. A subclonal CNA on chromosome 16 (indicated by black arrows) that was clearly visible in the tumor biopsy (top) became detectable in the matched cfDNA sample only after in silico size selection. **c** CNA profiles (ichorCNA) of longitudinal cfDNA samples derived from the same patient (EwS_5) support the monitoring of somatic clonal evolution for individual patients. The filtered CNA profiles of samples collected at diagnosis and two subsequent relapses are shown. The day of sample collection relative to the day of diagnosis is indicated (left). Inferred chromosomal gains are shown in red, inferred deletions are shown in green, and CNA-neutral regions are shown in blue.

First, we assessed how well cfDNA-derived CNA profiles recapitulate those of the corresponding primary tumor. To that end, we performed low-coverage whole-genome sequencing on DNA extracted from 43 matched EwS tumor samples (Supplementary Data 4). We generally observed high concordance between the CNA profiles of cfDNA and those of the matched primary tumors (Fig. 3a). However, we also identified individual cases in which the cfDNA-derived profiles showed CNAs that were not detected in the primary tumor. This can occur when the sequenced tumor sample (which comprises only a fraction of the entire tumor mass) does not include certain subclones that are detectable in cfDNA. We also observed cases in which specific CNAs were detected only in the tumor sample, but not the cfDNA. This may arise when certain subclones do not shed DNA into the bloodstream at high enough rates to be detectable. In four instances, in silico size selection for fragments in the range of 90–150 bp improved the detection threshold and confidence for specific CNA events in cfDNA (Fig. 3b).

Second, we compared the CNA-based quantification of tumor content between the unfiltered cfDNA sequencing data and the same data after in silico size selection for fragments in the range of 90–150 bp. Across the entire cfDNA data set, fragment-size filtering resulted in a mean increase of 19 percentage points for the inferred content of tumor-derived DNA (*p* < 0.001, Wilcoxon signed-rank test; Supplementary Fig. 2a and Supplementary Data 2). As the result of fragment-size filtering, we were able to identify several weak and often subclonal CNAs. Focusing on CNAs that are commonly found in EwS tumors (gains of Chromosome 1q, Chromosome 8, and Chromosome 12; deletion of Chromosome 16q)^48^, in silico size selection enhanced CNA detection in 11 cfDNA samples (Fig. 3b and Supplementary Fig. 2b).

Third, we explored the utility of the refined CNA-based analysis of cfDNA for minimally invasive monitoring of somatic evolution and disease progression. We focused on 13 patients with at least two cfDNA samples collected at different time points and more than 5% tumor-derived DNA content (according to ichorCNA). Based on the fragment-size filtered CNA dynamics we identified three groups of patients: those with a stable CNA profile over time (*n* = 5); those that exclusively lose (*n* = 1) or gain (*n* = 2) CNAs over time; and those that simultaneously lose and gain CNAs over time (*n* = 5) (Fig. 3c and Supplementary Fig. 2c). These results illustrate how EwS tumors follow diverse evolutionary dynamics over the course of diagnosis, treatment, and relapse.

In summary, we found that fragment-size filtering increases the sensitivity for detecting EwS-specific CNAs in cfDNA, allowing us to follow individual patients during disease progression with high subclonal resolution.

### Differences in cfDNA fragmentation along the genome reflect Ewing sarcoma-specific chromatin profiles

**Fig. 4:**
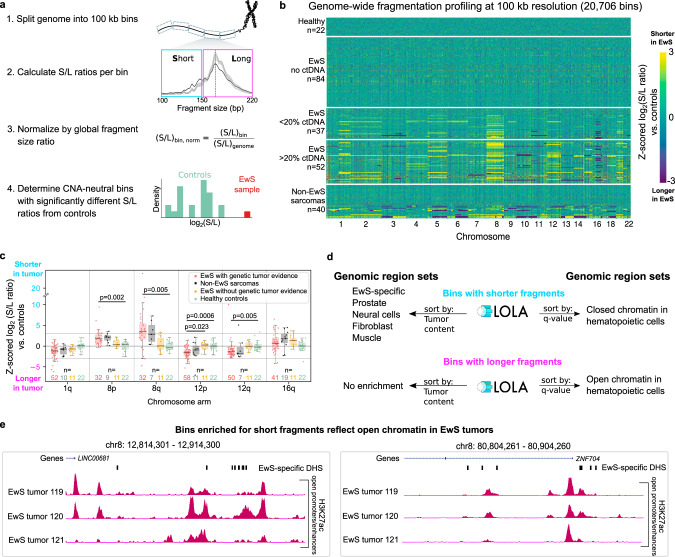
Regional fragment-size analysis detects an EwS tumor-specific epigenetic signature in cfDNA samples. **a** Schematic illustration of the regional fragment-size analysis, measuring the ratio of short (S) versus long (L) cfDNA fragments in 100 kb bins along the genome. Genomic regions that overlap with CNAs are excluded in order to focus the analysis on epigenetic signatures reflected in the cfDNA fragmentation patterns. **b** Heatmap comparing the genome-wide fragmentation profiles of cfDNA samples from patients with pediatric sarcoma to those of healthy controls. In each 100 kb bin (*n* = 20,706 bins), the log_2_(S/L ratio) of each sarcoma sample was compared to the distribution of log_2_(S/L ratios) of healthy controls via *z*-scores. Both CNA-affected and CNA-neutral bins are shown. EwS samples are grouped by genetically inferred tumor-derived DNA content. **c** Regional cfDNA fragmentation in patients with pediatric sarcoma compared to healthy controls. Only chromosome arms that are recurrently affected by CNAs in EwS tumors are shown. Box plots illustrate *z*-scores for EwS samples with genetic tumor evidence and without detected CNAs on the chromosomal arm (red), non-EwS sarcomas with genetic tumor evidence and without detected CNAs on the chromosomal arm (black), EwS samples without genetic tumor evidence (yellow), and healthy controls (green). The significance of the first group versus each of the other three groups was assessed using the two-sided Mann–Whitney *U* test; Bonferroni-corrected *p*-values are shown. Boxes correspond to interquartile ranges (IQR), thick black lines indicate the median, and the whiskers extend to the lowest or highest data point that are still within 1.5 IQR of the bottom or top quartile, respectively. **d** Functional enrichment analysis for regions with significantly shorter/longer cfDNA fragment size compared to healthy controls based on the LOLA software^54^. A selection of enriched terms is shown, while the full list is provided in Supplementary Data 6. **e** EwS tumor-specific epigenome profiles for selected regions with significantly shorter cfDNA fragment size compared to healthy controls. The genome browser profiles show open chromatin-associated histone H3K27 acetylation (for regions with shorter fragments) based on ChIP-seq data for primary EwS tumors^41^. EwS-specific DHSs along the selected genomic region are also indicated.

To investigate how the fragmentation patterns of tumor-derived cfDNA are influenced by the characteristic chromatin structure of EwS tumors, we analyzed the size distribution of cfDNA fragments in a position-dependent manner along the genome (Fig. 4a). We split the genome into 100 kb bins and calculated, for each bin, the ratio of short (S, 100–150 bp) to long fragments (L, 151–220 bp), resulting in genome-wide fragmentation profiles for each cfDNA sample^32,53^. The profiles of S/L ratios throughout the genome were normalized in each cfDNA sample and compared to the healthy controls (Fig. 4b).

We found that cfDNA samples with detectable tumor-derived DNA showed differential fragmentation patterns across entire chromosome arms; importantly, these differences persisted after excluding chromosomal arms harboring CNAs (individually for each sample), which have the potential to bias the S/L ratios (Fig. 4c and Supplementary Fig. 3a, b, Supplementary Data 5). For instance, fragments mapping to Chromosome 8 (which is commonly affected by chromosomal gains in EwS), were significantly shorter in the EwS cfDNA samples (higher S/L ratio). On the other hand, Chromosome 12 (which is also affected by recurrent chromosomal gains^48^) was significantly enriched in longer fragments (lower S/L ratio). Chromosome 1q (which is commonly affected by chromosomal gains) and Chromosome 16q (which is commonly affected by chromosomal deletions) were not significantly enriched for shorter or longer fragments (Fig. 4c and Supplementary Fig. 3b). Interestingly, the observed regional fragmentation patterns were similar between EwS and other pediatric sarcomas (Fig. 4c and Supplementary Fig. 3b). These results suggest that the observed fragmentation patterns cannot be explained as a side effect of CNAs but rather reflect different biological properties of these chromosomes.

To connect these observations to EwS biology, we performed region-set enrichment analysis (using the LOLA software^54^) on those CNA-neutral 100 kb bins that had significantly different S/L ratios compared to healthy controls (Fig. 4d and Supplementary Data 6). Based on LOLA’s comprehensive database of region sets with epigenetic and transcription-regulatory annotations, we found that bins with shorter fragments (higher S/L ratios) than in healthy controls were enriched for regions with EwS-specific open chromatin, showing peaks of promoter/enhancer-associated histone H3K27 acetylation in EwS tumors and EwS-specific DNase I hypersensitive sites (Fig. 4e and Supplementary Data 6). In contrast, none of the non-EwS tumor samples or randomized bins showed enrichment for this genomic region set, thus confirming the specificity of the observed EwS signature.

Bins with short fragments in EwS cfDNA samples were also enriched for regions of open chromatin in the prostate, neural cells, fibroblasts, and muscle (Fig. 4d)—cell types that share key biological properties with EwS cells and/or with the suspected cell-of-origin of EwS^45^. Moreover, they were enriched for regions of closed chromatin in hematopoietic cells, while bins with lower S/L ratios than in healthy controls (i.e., longer fragments in EwS cfDNA samples) were enriched for open chromatin in hematopoietic cells. These results are consistent with evidence that cfDNA in healthy donors is primarily derived from hematopoietic cells^2,35,55^, and it is very likely that the tumor-derived DNA in cfDNA samples from EwS patients results in a lower ratio of blood-derived cfDNA.

Finally, we tested if regional fragmentation patterns could be used for patient-specific disease monitoring in EwS, in order to complement the CNA-based analysis described above (Fig. 3c and Supplementary Fig. 2c). We focused on those seven patients for whom we had cfDNA samples with genetic tumor evidence both at diagnosis and relapse, and we selected CNA-neutral genomic bins overlapping with regions of EwS-specific open chromatin. Among the bins that showed variable S/L ratios over time, two gene loci with a well-established role in EwS stood out (they were ranked fifth and sixth overall): *STAG1*^56^ and *SMARCC1* (*BAF155*)^57^ (Supplementary Fig. 3c, d and Supplementary Data 7). Although this analysis requires further validation, especially in matched tumor samples collected at diagnosis and relapse, it illustrates the potential of liquid biopsies for monitoring the state of gene-regulatory elements during disease progression.

In summary, we found that regional differences in cfDNA fragmentation across the genome reflect the chromatin structure of the EwS tumor cells—and of the normal hematopoietic cells—that contribute to the cfDNA circulating in the blood stream.

### cfDNA fragmentation at EwS-regulatory regions detects tumor-derived DNA independent of genetic alterations

**Fig. 5:**
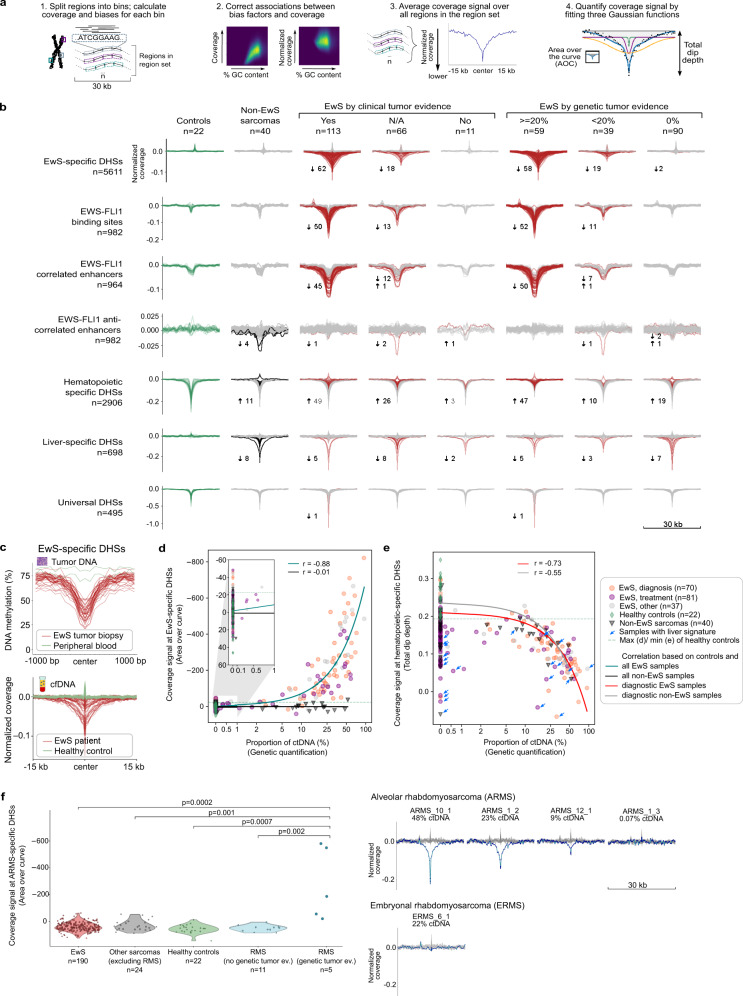
Fragment analysis for EwS-specific genomic regions quantifies tumor-derived cfDNA in EwS patients. **a** Conceptual outline of the LIQUORICE method and software for fragment analysis of cfDNA based on tumor-specific epigenetic alterations. **b** Aggregated, bias-corrected, and normalized coverage signals at selected genomic region sets shown for healthy controls, for non-EwS sarcomas, and for EwS cfDNA samples. EwS samples are grouped by genetically inferred tumor-derived DNA content and clinical tumor evidence. cfDNA samples with coverage signals significantly different (three standard deviations) from healthy controls are displayed in red; the total number of those samples and the direction of the deviation (arrow) are indicated. Total dip depth was used as the metric of choice for the sharp dips at hematopoietic-specific and universal DHSs; area over the curve (AOC) was used for the other region sets. **c** cfDNA-based coverage signal at EwS-specific DHSs (bottom, *n*_EwS_ = 38) reflects the aggregate DNA methylation profiles at these regions in matched tumor biopsies (top, *n*_EwS_ = 38). **d** Scatterplots showing the correlation of the coverage signal at EwS-specific DHSs with the genetically inferred tumor-derived DNA content of the cfDNA samples. Pearson correlation coefficients (*r*) and linear trend lines are shown. The x-axes are shown in a log scale from 1% onwards. **e** Same as **d** but showing the coverage signal at hematopoietic-specific DHSs. Blue arrows indicate samples with significant liver signature. **f** Aggregated, bias-corrected, and normalized coverage signal (AOC) at alveolar rhabdomyosarcoma (ARMS)-specific DHSs for cfDNA samples from healthy controls and patients with EwS, RMS, and other pediatric sarcomas (left; *p*-values were calculated using two-sided Mann–Whitney *U* tests without correction for multiple testing). For ARMS patients with at least 9% ctDNA (genetic-based evidence), a striking reduction of fragment coverage was observed (right). A cfDNA sample from a patient with embryonal rhabdomyosarcoma (ERMS) did not show any reduction of fragment coverage at ARMS-specific DHSs (bottom right).

Building upon our observation that the characteristic chromatin structure of EwS is detectable in the fragmentation patterns of cfDNA, we explored the feasibility of monitoring tumor-derived DNA independent of any genetic alterations. To that end, we developed a dedicated method and software tool for fragmentation analysis of cfDNA in the context of tumor-specific epigenetic alterations—such as the characteristic regions of de novo open chromatin that we and others previously discovered in EwS^41,58,59^. Our new tool, which we named LIQUORICE (for liquid biopsy regions-of-interest coverage estimation), overlays genome-wide cfDNA fragment profiles with a predefined set of genomic regions that are frequently altered in the studied cancer type, and it calculates a bias-corrected consensus (composite) signature of fragment coverage throughout these regions-of-interest (Fig. 5a and Supplementary Fig. 4a, b).

We focused on four types of genomic region sets with previously reported regulatory relevance in EwS^41,59^: (i) EwS-specific DNase I hypersensitive sites (DHSs); (ii) EWS-FLI1 binding sites; (iii) EWS-FLI1-correlated enhancers, defined as elements that lose histone H3K27 acetylation upon EWS-FLI1 knockdown; and (iv) EWS-FLI1-anti-correlated enhancers, defined as elements that gain histone H3K27 acetylation upon EWS-FLI1 knockdown (Supplementary Data 8). For each cfDNA sample and each region set, we determined the consensus signature of cfDNA fragment coverage by averaging across all regions of the given type (Fig. 5b). For patients with EwS (especially those with detectable tumor-derived DNA), we observed a striking reduction of cfDNA fragment coverage for EwS-specific DHSs, EWS-FLI1 binding sites, and EWS-FLI1-correlated enhancers. In contrast, EWS-FLI1-anti-correlated enhancers showed no such depletion, nor did cfDNA samples from other pediatric sarcomas, whereas universally open DHSs showed similar depletion patterns in all patients and in healthy individuals. These results emphasize that the focus on regions with EwS-specific open chromatin confers specificity regarding the tumor type to our LIQUORICE-based analysis of cfDNA fragmentation patterns.

To confirm that the depletion of cfDNA fragments at the EwS-regulatory regions indeed reflects the characteristic epigenetic states of the tumors from which the cfDNA is derived, we performed genome-scale DNA methylation profiling in matched tumor samples (*n* = 38), using the reduced representation bisulfite sequencing assay^41^ (Supplementary Data 4). We plotted the mean DNA methylation levels across EwS-specific DHSs and observed a striking depletion of DNA methylation in those regions in the primary tumors, mimicking the depletion of cfDNA fragments (Fig. 5c). This result is consistent with our previous finding that DNA methylation in primary EwS tumors is depleted at EwS-specific DHSs^41^, and it provides further support that the observed fragmentation patterns in cfDNA are indeed the result of the characteristic chromatin structure in primary EwS tumors.

To quantify the reduction (dip) of fragment coverage at EwS-specific regulatory regions, we fitted three Gaussian functions to the bias-corrected consensus signature, and we calculated the dip area (i.e., area over the fitted curve, AOC) and dip depth for each cfDNA sample (Fig. 5a and Supplementary Fig. 4c, and Supplementary Data 9). These two scores reflect the sample-specific regulatory activity of the selected region set: large areas and high depths indicate strong depletion of fragments at EwS-specific regulatory regions, and a high proportion of tumor-derived DNA in the corresponding cfDNA sample. The EwS-specific coverage signal strongly correlated with genetically inferred tumor content (Pearson *r* = 0.88) (Fig. 5d), indicating that coverage at EwS-specific DHSs may be useful for quantifying tumor-derived DNA content independent of genetic alterations.

In total, 80 cfDNA samples obtained from 54 patients with EwS showed significantly reduced fragment coverage around the EwS-specific DHSs compared to healthy controls (|*z*-score| > 3). For 62 of these 80 cfDNA samples, the clinical data supported the presence of a tumor at the time of cfDNA sample collection, and in 17 out of the remaining 18 samples, the identification of tumor-derived DNA by LIQUORICE was supported by genetic evidence (detection of CNAs and/or gene fusion). When we grouped our samples by genetically inferred tumor content, 58 out of 59 samples with >20% genetic tumor content had significantly reduced coverage at EwS-specific DHSs, while this number dropped to 19 out of 39 samples with genetic tumor content in the range of 0.1–20% and to 2 out of 90 samples with 0% genetic tumor content (Fig. 5b).

We also used LIQUORICE to quantify the contribution of non-tumor cells to the cfDNA samples (Supplementary Data 8). We focused specifically on open chromatin regions in hematopoietic cells (as the main source of cfDNA in healthy controls^2,35,55^) and liver tissue (as a proxy of chemotherapy-induced organ damage). For regions with hematopoietic open chromatin, we observed strongly reduced fragment coverage in healthy controls, whereas the reduction was much weaker for patients with EwS (Fig. 5b). The hematopoietic-specific coverage signal strength correlated negatively with the genetic estimate of tumor-derived DNA in the cfDNA sample (Pearson *r* = −0.73) (Fig. 5e and Supplementary Fig. 5a, b). For regions with liver-specific open chromatin, we observed strongly reduced fragment coverage in a subset of those patients who received chemotherapy at the time of sample collection, which correlated with serum-based protein markers of liver damage (Fig. 5e and Supplementary Fig. 5c and Supplementary Data 1). High proportions of liver-derived DNA also explained most cases in which the coverage signatures of tumor-derived DNA and of hematopoietic DNA were simultaneously low (Fig. 5e and Supplementary Fig. 5d).

Finally, we tested whether the fragmentation analysis of tumor-specific region sets could be generalized to other types of pediatric sarcoma. We focused on alveolar rhabdomyosarcoma (ARMS), a pediatric sarcoma which in most cases, similarly to EwS, is driven by an oncogenic fusion protein (PAX3-FOXO1)^60^. Applying our LIQUORICE software on a set of ARMS-specific DHSs that we defined using publically available data^61^ (Supplementary Data 8), we indeed observed a characteristic reduction in cfDNA fragment coverage that was specific to ARMS samples (Fig. 5f). This analysis confirms that the LIQUORICE analysis of cfDNA fragmentation patterns generalizes beyond EwS, and it suggests that our method might be broadly useful for detecting and quantifying tumor-derived DNA independent of any genetic aberrations.

In summary, we developed a method and software that measures tumor-derived cfDNA based on fragmentation patterns that reflect the chromatin structure of the primary tumor. We also demonstrated quantitative monitoring of cfDNA derived from other tissue types including hematopoietic cells (negatively correlated with tumor content) and liver (indicative of organ damage), and we showed that our method generalizes beyond EwS.

### Non-genetic fragmentation-based methods improve the accuracy and robustness of liquid biopsy analysis in EwS

In the final part of our analysis, we assessed whether fragmentation-based methods can improve the identification and classification of patients with EwS compared to conventional liquid biopsy analysis based on genetic alterations. As input for our benchmarking, we used the full range of metrics provided by each of the three fragmentation-based methods introduced above. Moreover, we included read depth in five megabases (Mb) bins as an additional input that reflects CNAs. We then trained four machine learning classifiers (support vector machine, neural network, random forest, and generalized linear model with elastic-net regularization) for each of the four feature sets, and we evaluated their performance using cross-validation in 100 iterations of bootstrapping. We additionally constructed a meta-learner, which weighted and combined the predictions of the individual classifiers that were based on single feature sets. Importantly, all performance metrics were calculated on unseen test sets, and the configuration of the analysis avoided potential information leakage that could result in overtraining.

**Fig. 6:**
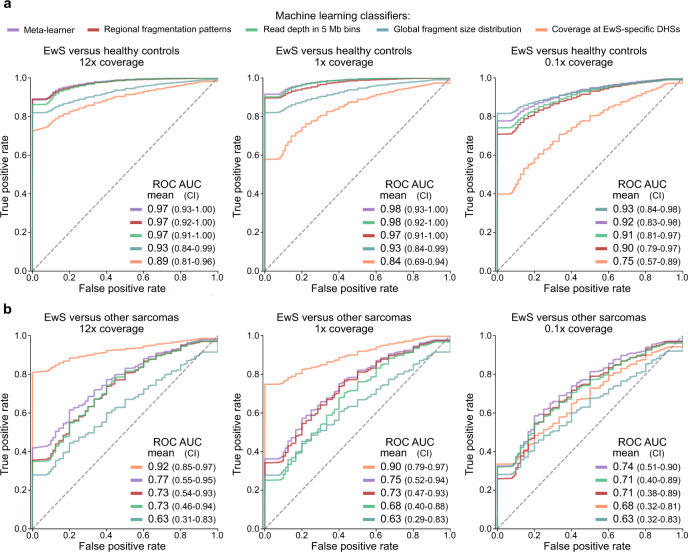
Fragment-based analysis of cfDNA enables accurate tumor detection and classification. Prediction performance of machine learning classifiers trained to distinguish patients with EwS from healthy controls (**a**) and from patients with other pediatric sarcomas (**b**), based on the following sets of input features: global fragment-size distribution (blue); fragment coverage at EwS-specific DHSs (orange); read depth in 5 Mb bins (green); and regional fragmentation patterns (red). Results are also shown for a meta-learner combining the predictions of all individual classifiers into a weighted consensus prediction (purple). The performance of each model was evaluated by and averaged over 100 iterations of bootstrapping, separately for the different sequencing coverage levels (median of 12×, 1×, and 0.1×). CI is the 95% confidence interval obtained by bootstrapping. **a** ROC curves show, for each feature set, the performance for distinguishing between cfDNA samples from patients with clinical evidence for EwS (*n*_samples_ = 103) and healthy controls from three independent sets (22 controls sequenced in this study; 22 controls from Cristiano et al.^32^; and 24 controls from Ulz et al.^34^). Machine learning models were trained separately for each of the 3 control sets; the mean results over the 3*100 bootstrap iterations are shown. **b** ROC curves show the performance of each feature set for distinguishing between cfDNA samples from patients with EwS (*n*_samples_ = 98) and from patients with other pediatric sarcomas (*n*_samples_ = 18). For both sets of samples, we ensured the presence of tumor-derived cfDNA in the blood based on genetic evidence.

First, we evaluated the machine learning classifiers for their ability to distinguish between cfDNA from patients with clinical evidence of EwS tumor presence (103 samples from 73 patients) and cfDNA from healthy individuals. Given that our cohort comprised only 22 healthy individuals, we incorporated 46 additional control samples from two published data sets^32,34^, which we normalized to make them comparable to our data set (Supplementary Fig. 6a). We observed excellent prediction performance for distinguishing between cfDNA from patients with EwS and cfDNA from healthy individuals, with receiver operating characteristic (ROC) area under the curve (AUC) values of up to 0.97 (Fig. 6a and Supplementary Data 10). The prediction performance was highly similar independent of which set of healthy individuals was used as controls (Supplementary Fig. 6b) and which machine learning algorithm was used (Supplementary Fig. 6c–g). We further validated our machine learning analysis by confirming its lack of predictiveness on randomly shuffled labels (Supplementary Fig. 6h), and by including only diagnostic EwS cfDNA samples (one per patient) in the analysis (*n* = 64) (Supplementary Fig. 6i).

Given that deep whole-genome sequencing of cfDNA is costly as a routine diagnostic assay, we systematically evaluated the prediction performance not only at our full coverage (~12×), but also for down-sampled coverage of 1× and 0.1×. We found that the meta-learner, as well as the classifiers based on read depth and the regional fragment-size distribution, profited most strongly from deep whole-genome sequencing, and at 0.1× coverage, they were outperformed by using only the global fragment-size distribution. Indeed, the latter classifier was entirely unaffected by the coverage reduction, achieving ROC AUC values of 0.93 at all sequencing depths (Fig. 6a).

We also compared our machine learning classifiers to established genetic methods for the detection of tumor-derived cfDNA, namely CNA quantification using ichorCNA and fusion gene detection using either whole-genome sequencing or ddPCR. We found that the meta-learner achieved higher sensitivity at 100% specificity (i.e., using a threshold that correctly classified all healthy individuals in the test set) than conventional genetic methods, independent of whether we included all samples (*n* = 103, 85% versus 65% sensitivity) or only those for which all three genetic measurements were possible (*n* = 56, 90% versus 79% sensitivity) (Supplementary Fig. 7).

Fragment coverage at EwS-specific DHSs correlated well with the genetic methods (Supplementary Figs. 8 and 9) and showed a similar association with clinical annotations (Supplementary Fig. 10). This observation supports the feasibility of substituting genetic methods by fragment coverage at tumor-specific regulatory regions for cancers with few genetic aberrations. We also observed that the detection of tumor-derived cfDNA based on the coverage signal at EwS-specific DHSs in patients with localized EwS (*n* = 45) was associated with shorter relapse-free survival (RFS, *p* = 0.005, log-rank test) as well as shorter overall survival (OS; *p* = 0.034, log-rank test). For RFS, this association remained significant in a multivariate analysis (*p* = 0.042, Wald test). The associations with survival were somewhat more pronounced for the quantification of tumor-derived DNA based on coverage at EwS-specific DHSs than for the genetic methods (Supplementary Fig. 11).

Finally, we tested how well each of the classifiers could distinguish EwS from other sarcomas, a task with high clinical relevance^62,63^. Focusing on patients with genetic evidence of tumor-derived cfDNA (EwS: 89 samples from 51 patients; other sarcomas: 18 samples from 14 patients), fragment coverage at EwS-specific DHSs outperformed all other metrics at high and medium sequencing coverage, reaching a ROC AUC value of 0.93 at high sequencing coverage (Fig. 6b). This fragment-based method also classified EwS more sensitively than detection of the *EWS-Ets* fusion gene by whole-genome sequencing (81% versus 73% sensitivity at 100% specificity) (Supplementary Data 2 and 10). These results illustrate the feasibility of LIQUORICE-based analysis of cfDNA fragment coverage for the differential diagnosis of epigenetically distinct tumor types using liquid biopsies.

In summary, we developed machine learning classifiers that leverage fragment-based methods for accurate distinction between patients with EwS and healthy individuals, and between patients with EwS and other sarcomas, thereby establishing a method for liquid biopsy analysis that does not depend on recurrent genetic aberrations.

## Discussion

We present a comprehensive genetic and epigenetic analysis of cell-free DNA in Ewing sarcoma, with the goal of widening the scope and applicability of liquid biopsies in pediatric oncology. We show that whole-genome sequencing of cfDNA (when combined with suitable computational methods, some of which we developed here) provides a one-size-fits-all assay for liquid biopsy analysis, allowing us to: (i) detect tumor-derived DNA with high sensitivity and without requiring any somatic mutations or CNAs; (ii) distinguish between different cancer types based on their characteristic epigenetic signatures; (iii) monitor CNAs and disease progression over time; (iv) assess treatment-induced toxicity and organ damage based on cfDNA released from dying cells; and (v) estimate survival and relapse probabilities at diagnosis. Importantly, our approach is practically feasible in a clinical setting, requires less than 10 ng of cfDNA, profits from falling sequencing costs, does not require access of primary tumor tissue, and is informative even in the absence of any genetic alterations.

Our work builds upon previous studies of fragmentation patterns in cfDNA^5,32–35,49,50,53,64,65^, which we extended in several ways to enable an integrated genetic and epigenetic analysis of EwS tumors based on liquid biopsies. First, by analyzing the global fragment-size distribution of cfDNA in a sarcoma cohort, we showed that tumor-derived DNA in pediatric solid tumors follows similar fragmentation patterns as observed in adult cancers, which enabled robust quantification of tumor content even at reduced sequencing coverage (down-sampled from ~12× to 0.1×). Second, our analysis of the regional fragment-size distribution along the genome uncovered evidence of tumor-specific epigenetic alterations, which supported sensitive and specific identification of cfDNA samples that contained tumor-derived DNA. Third, we developed and validated a dedicated method and open source software (LIQUORICE) for assessing fragment coverage at regions-of-interest with characteristic epigenetic changes in the tumor and in other sources of cfDNA such as blood and liver. This method enabled the distinction between different tumor types based on their epigenetic profiles as well as the accurate quantification of tumor-derived cfDNA independent of genetic aberrations. Fourth, we showed that machine learning classifiers exploiting these patterns achieve accurate tumor detection and classification in our cohort, outperforming conventional genetic analysis based on CNAs and fusion gene detection by whole-genome sequencing or ddPCR. Fifth, we found that the fragment-based cfDNA metrics may have prognostic value in pediatric sarcomas, given that a negative association between the detection of tumor-derived cfDNA and patient survival was observed.

Our study provides one of the largest whole-genome sequencing-based analyses of cfDNA in any childhood cancer and a broadly useful resource for advancing the use of liquid biopsies in pediatric oncology. Nevertheless, the following limitations of the current study should be considered by researchers building on our results. First, the lack of gold standards for quantifying tumor-derived cfDNA that could be used as a reference makes it difficult to provide definitive performance metrics for the machine learning classifiers. To mitigate this potential concern, we used clinical evidence (mainly based on radiological imaging) as well as three lines of genetic evidence (CNAs detected by ichorCNA, fusion genes detected by whole-genome sequencing, and by ddPCR) as our reference. Second, even among pediatric cancers, the sarcomas that we investigated here are relatively rare (EwS accounts for ~2% of cancers diagnosed in children and adolescents^45^), which limited the size of the cohort and required the combination of samples from several centers and countries. Third, the analysis was conducted in a retrospective manner and was not embedded in a dedicated clinical trial. Validation in a large, prospective study cohort will be required to confirm the clinical associations and to qualify the method for routine clinical use.

In summary, our study demonstrates how deep whole-genome sequencing of cfDNA enables comprehensive detection, classification, and monitoring of pediatric tumors based on their genetic and epigenetic profiles, thus providing a clinically relevant method for liquid biopsy analysis in cancers with few or no genetic alterations.

## Methods

### Patient cohort

This study included 200 plasma samples from 95 patients with EwS and 41 plasma samples from 31 patients with other types of sarcoma: EwS-like sarcoma (3 patients, two of which were positive for the CIC-DUX4 fusion gene), osteosarcoma (8 patients), rhabdomyosarcoma (12 patients), synovial sarcoma (3 patients), and other types of sarcoma (5 patients) (Supplementary Data 1). Plasma samples from 22 healthy individuals (24–50 years old) were used as controls and were recruited for this study (7 individuals) or obtained via the Austrian Red Cross (15 individuals). In total, we analyzed 263 plasma samples obtained from the following institutions: St. Anna Kinderspital, Vienna, Austria (55 samples); St. Anna CCRI biobank, Vienna, Austria (35 samples); Red Cross, Vienna, Austria (15 samples); Institute Curie, France (25 samples); University Hospital Erlangen, Germany (99 samples); and Oslo University Hospital, Norway (34 samples). We also obtained 43 tumor biopsies (22 fresh frozen tissues; 21 FFPE tissues) from 42 of the patients for which plasma samples were available (Supplementary Data 4). Of these tumor samples, one was collected at relapse, while all others were collected at the time of diagnosis. Most of the patients with EwS included in this study were treated according to the EWING2008 protocol or slight variations of it^66^. Patients from Norway were treated according to the ISG/SSG III protocol^67^. All samples were obtained with informed consent and with approval by the following review boards: Ethics Committee of the Medical University of Vienna (1292/2018), CPP SUD-EST IV, CPP 14/070, EE2012 study (reference number A 14-419), CPP ILE DE FRANCE III, CPP 3272, MAPPYACT study (reference number 2015-A00464-45), CPP ILE DE FRANCE IV, CPP 56-14, NGSKids study (reference number 2014-A00701-46), Ethics Committee of the “Ärztekammer Westfalen-Lippe und der Westfälischen Wilhelms-Universität Münster” (2008–391-f-A; EudraCT 2008–003658-13 EWING2008), and Ethics Committee for Medical Research in Southeastern Norway (17866). Clinical data for the patients included in this study are provided in Supplementary Data 1.

### DNA isolation

Plasma samples from Germany were prepared as follows: Blood samples were collected in EDTA tubes and centrifuged within 2 h at 1200×*g* for 10 min. Plasma was separated from peripheral blood cells, aliquoted into microtubes, and frozen at −80 °C. cfDNA was isolated using the QIAsymphony Circulating DNA Kit with the QIAsymphony SP (Qiagen) instrument or the QIAampMinElute cfDNA Kit (Qiagen) for manual isolation according to the manufacturer’s recommendations^23^. Plasma samples from France were obtained in EDTA tubes and prepared by centrifugation at 2000 rpm for 10 minutes within 1–24 h after collection^20^. cfDNA was extracted using the QIAamp Circulating Nucleic Acid Kit (Qiagen) with the Qiavac24s system, according to the manufacturers’ recommendation. For the St. Anna CCRI biobank plasma samples, a cell stabilization step using formaldehyde was implemented during plasma preparation^68,69^. For all other plasma samples, cfDNA was isolated as follows: Whole blood was collected in EDTA tubes and processed within a few hours. Plasma and cellular components were separated by centrifugation at 1600×*g* for 10 min with gentle break and acceleration set to 1. Plasma was centrifuged a second time for 10 min at 16,000×*g* at room temperature to remove any remaining cellular debris and stored at −80 °C until the time of cfDNA extraction. cfDNA was isolated from plasma (0.2–4.8 ml) using the QIAamp Circulating Nucleic Acid Kit (Qiagen) and eluted in 45 µl elution buffer using DNA LoBind tubes (Eppendorf). The concentration of cfDNA was determined by the Qubit dsDNA HS Assay Kit (ThermoFisher Scientific). The amount of plasma used per sample and the corresponding cfDNA concentrations are provided in Supplementary Data 1. Tumor DNA was isolated from snap-frozen tumors and formalin-fixed, paraffin-embedded (FFPE) tumor tissues by standard proteinase K digestion and phenol/chloroform extraction^41^. DNA was quantified using a Qubit 2.0 Fluorometer (ThermoFisher Scientific, Q32866) and the Qubit dsDNA BR Assay Kit (ThermoFisher Scientific, Q32850).

### Library preparation and sequencing

Whole-genome sequencing libraries were generated from 10 ng of cfDNA unless noted otherwise (Supplementary Data 1), using the NEBNext Ultra II DNA Library Prep Kit for Illumina (New England Biolabs). Briefly, cfDNA was processed without further fragmentation or size selection, amplified and barcoded after adapter ligation with 6–10 PCR cycles (Supplementary Data 1), depending on a qPCR amplification check. Cleanups were performed with AMPureXP beads (Beckman Coulter) with a 1.2× volume ratio. Final libraries were eluted in 20 µl nuclease-free water, quantified with the Qubit dsDNA HS Assay Kit (Supplementary Data 1), and the profile was checked on a TapeStation 4200 (Agilent Technologies). Libraries for 15 French samples were prepared without fragmentation using the Kapa Library Preparation Kit for Illumina platforms (Kapa Biosystems)^20^ and included a size selection step before sequencing. These samples were excluded from the analysis corresponding to Figs. 2, 4, and 6, and from the associations with clinical data. Formaldehyde-fixed samples that showed signs of affected epigenetic properties (*n* = 11, CCRI Biobank; Supplementary Data 2), were excluded from all epigenetics-based analyses (i.e., Figs. 2, 4, 5, 6, and clinical associations). Low-coverage whole-genome sequencing (lcWGS) libraries for tumor DNA were generated as described above for cfDNA samples, with an additional shearing step. For shearing, a Covaris M220 device was used with MicroTUBE-50 AFA Fiber Screw-cap tubes (Covaris) and the following settings: 75 peak incident power, 10% duty factor, 200 cycles per burst, 90 s at room temperature. Reduced representation bisulfite sequencing (RRBS) libraries for tumor DNA were generated as described previously^41^. Tumor DNA amounts used for each assay are specified in Supplementary Data 4. cfDNA and lcWGS libraries were sequenced on a NovaSeq 6000 machine using NovaSeq S4 2 × 100 bp flowcells for cfDNA and 2 × 50 bp flowcells for lcWGS. In addition, pilot experiments for 18 cfDNA samples were performed using Illumina HiSeq 3000/4000 machines. RRBS libraries were sequenced on Illumina HiSeq 3000/4000 machines with 2 × 50 bp flowcells.

### Whole-genome sequencing data processing

Base calls provided by the Illumina Realtime Analysis software were converted into BAM files using Illumina2bam (https://github.com/wtsi-npg/illumina2bam) and demultiplexed using BamIndexDecoder from the same package. Initial quality control was performed using the FastQC software (http://www.bioinformatics.babraham.ac.uk/projects/fastqc/). Adapter trimming, initial quality control, and read-level filtering were performed with fastp^70^ using default settings. Next, quality-filtered reads were mapped to hg38 using the BWA-MEM software^71^ with default settings. Samblaster^72^ was used to mark duplicates, which were subsequently removed. All bioinformatic analyses were relative to the GRCh38/hg38 assembly of the human genome. A summary of the sequencing statistics is provided in Supplementary Data 1 and 4.

### RRBS data processing

Bisulfite sequencing data were processed as follows^41^: Read sequences were trimmed using Trimmomatic with the following settings: ILLUMINACLIP: RRBS_adapters.fa:2:40:7 SLIDINGWINDOW:4:15 MAXINFO:20:0.50 MINLEN:18. Reads were aligned to the GRCh38 assembly of the human genome, using BSMAP in its RRBS mapping mode. DNA methylation levels for individual CpGs were calculated using custom Python scripts. Bisulfite conversion efficiency was estimated by aligning unmapped reads to the spike-in genome for methylated or unmethylated control sequences. CpGs located in repetitive regions according to the UCSC RepeatMasker track were excluded from further analysis. Mean DNA methylation levels across EwS-specific DHSs were quantified and plotted using the MIRA v1.8.0^73^ with the following settings: region size was set to 2000 bp, number of bins per region was set to 21, minBaseCovPerBin was set to 100, and the center of the 2000 bp regions was used for plotting.

### EWS-Ets fusion gene detection using whole-genome sequencing data

Aligned BAM files were loaded into the IGV software^74^, and the relevant genomic regions (*EWSR1*, *FLI1*, and *ERG* genes) were manually screened for a cluster of discordant reads indicating translocation to one of the potential fusion partners. In addition, reads with a significant portion of mismappings only on one side of the read were extracted to identify potential split reads where the breakpoint was near to the end of the read. Finally, paired-end reads supporting the translocation (each read mapped to the individual genes on different chromosomes) without including the breakpoint were also used as evidence of the gene fusion. This manual procedure proved more sensitive than automated methods, especially in cases with very few supporting reads. All putative translocation reads were further evaluated with BLAT^75^ to validate true split reads resulting in breakpoint coordinates and to identify any additional bases that might have been inserted during the repair process. In case of microhomologies directly at the breakpoint, their length was recorded. All identified breakpoint coordinates, the genes involved, the type of event (main, reciprocal, or complex), and a representative read at the breakpoint are provided in Supplementary Data 2.

Once breakpoint coordinates and possible microhomologies were identified, quantification of fusion reads and corresponding germline reads was performed, requiring at least one base clearly distinguishing the fusion from the germline (outside of the microhomology). To this end, we added the manually inferred *EWS-Ets* fusion sequence and the surrounding genomic region (plus/minus 500 bp) to the reference genome and used the resulting file as a reference for re-mapping of reads in the breakpoint region (both breakpoint coordinates plus/minus 1000 bp). We then counted the number of fragments that aligned to the fusion sequence and contained reads that spanned the breakpoint. This number was compared to the number of fragments with reads spanning the breakpoint coordinates at the germline sequences on both chromosomes. For both counts, we considered microhomologies and required that reads fully span them if they are detected. To reduce the technical variability due to coverage fluctuations and sequence composition biases around the breakpoints, germline counts for both involved fusion partners around the breakpoint were averaged. Tumor content based on breakpoints was calculated by using the formula 2*f/(((g_1_ + g_2_)/2)+f) (f: fusion gene, g: germline; Supplementary Data 2). Additionally, if more than one breakpoint was detected (for instance the main *EWS-FLI1* fusion, the reciprocal *FLI1-EWS* event, and/or multiple breakpoints in case of complex local rearrangements) the mean was used.

### EWS-Ets fusion gene quantification using ddPCR

Patient-specific assays for fusion gene detection and quantification were designed following the guidelines from the Bio-Rad ddPCR application guide bulletin 6407, using a double-fluorescently labeled probe (FAM-BHQ1) that crosses the genomic DNA breakpoint as well as two flanking primers. For normalization, a HEX-BHQ1 probe targeting an invariant region in the genome (on Chromosome 4, 9, or 10) was designed with a similar amplicon length as the fusion assay. The normalizer was chosen based on the CNA profile of each individual sample and required a region with a normal copy-number state. Primers and probes were ordered through Sigma-Aldrich (Merck); their sequences are provided in Supplementary Data 2. All reactions contained 900 nM of each primer, 250 nM of the mutant and normalizer probe, 2× ddPCR Supermix for Probes (no dUTP), and up to 7.8 µl of PE library as input in 22 µl total volume. We used whole-genome sequencing libraries as input (instead of cfDNA) due to the limited cfDNA amounts. Fluorescence signals were measured on a QX200 ddPCR system (Bio-Rad) and analyzed with the QuantaSoft software v1.7.4 (Bio-Rad). Genomic DNA from healthy peripheral blood mononuclear cells (PBMCs) was used as a negative control for fusion assays, and water served as a negative control for the fusion assays as well as the normalizer assays in each experiment. Events with more than three positive droplets were called positive, and their ddPCR-based tumor content was calculated by doubling the fusion counts and dividing this number by the normalizer counts, assuming a heterozygous fusion in the tumor cell (Supplementary Data 2).

### CNA analysis and quantification of tumor-derived DNA based on read depth

To determine CNAs and estimate tumor-derived DNA content, we used the ichorCNA tool^7^ (version from git commit 1d54a1f), which was specifically designed to work with cfDNA data. First, reads with mapping quality >20 were counted in 500 kb windows using the readCounter tool from the HMMcopy R suite (v1.2.0)^76^. Then, ichorCNA was run on the resulting WIG files. As no matched germline control samples were available, ichorCNA was set to use its internal set of reference samples to generate log_2_-ratios of sample versus control. Window size was set to 500 kb, the minimum recommended size for this application. Homozygous deletions were not allowed as a possible scenario, as it is recommended for large windows. In addition to CNA profiles, ichorCNA provides estimates of tumor-derived DNA content for different ploidy states, ranking them by log-likelihood. The minimum, maximum, and top-ranked tumor content values were recorded. After visual inspection and manual comparison of the suggested ploidy states in case for longitudinal samples, the most plausible tumor content value based on ichorCNA read depth was recorded and used for downstream analysis (Supplementary Data 2).

### Tumor content quantification based on combined genetic evidence

The individual values for the tumor content derived from whole-genome sequencing breakpoints, ddPCR, and ichorCNA read depth were used to infer a combined genetic estimate of the tumor-derived DNA content in each cfDNA sample (Supplementary Data 2).

### Global fragment-size distribution analysis

Fragment-size distributions were inferred from mapping coordinates of read pairs, using Picard CollectInsertSize-Metrics (v2.8.1; http://broadinstitute.github.io/picard/) with default settings apart from the histogram width parameter, which was set to 800. For further analysis, frequencies were then calculated relative to the total number of reads. For visualization purposes, frequencies were shown as the number of fragments with a specific size divided by the total number of fragments in the displayed size range. The effect of in silico size selection on the tumor content as estimated by CNAs was calculated as follows: We used all samples for which ichorCNA called the same number of copies for each chromosome for both the size-selected and non-size-selected input. The resulting matching tumor content estimates were recorded and used for significance testing with the Wilcoxon signed-rank test.

### Regional fragment-size distribution analysis

The genome was split into bins (tiling windows) with a length of 100 kb each using deeptools (deeptools suite v3.1.2)^77^, and the number of short (S, 100–150 bp) and long (L, 151–220 bp) fragments mapping to the bin was recorded. The bin size of 100 kb was chosen as a compromise between high genomic resolution and a large enough number of reads per bin for robust estimations of the S/L ratio (~25,000 expected reads per bin). Regions overlapping the ENCODE blacklist^78^ or the hg38 gap track (https://genome.ucsc.edu) were excluded (these regions tend to be badly mappable, and we preferred to use slightly fewer, but more reliable bins for our analysis). Subsequently, GC bias was corrected with LOWESS smoothing (considering 75% of the data for smoothing), separately for the short and long fragments. Using these corrected values, the log_2_(S/L ratio) was calculated as log_2_(number of short fragments/number of long fragments). This value was then normalized, subtracting the genome-wide log_2_ of the S/L ratio, which was calculated separately for each cfDNA sample by averaging over all bins for which ichorCNA (using in silico size-selected input for maximum sensitivity) indicated a CNA-neutral state. The same procedure, also using only bins for normalization that were called CNA-neutral in the sample of interest, was applied to the healthy control samples (*n* = 22). Then, the log_2_(S/L) value of each bin was compared to the distribution of control samples via *z*-scores, and bins were marked as significantly shorter or longer if the FDR-corrected *p*-value, based on these *z*-scores, was below 0.05. This procedure was also applied to the healthy control samples, for which the *z*-score was calculated relative to the distribution of all other healthy control samples (*n* = 21). Additionally, bins were marked as CNA-neutral or CNA-affected, again depending on ichorCNA’s output. Bins that were filtered by ichorCNA as unreliable regions (or for which ichorCNA could not determine the CNA state) were excluded from the analysis. For the chromosome arm analysis, the log_2_(S/L) was averaged for all bins within a chromosomal arm, and then compared to the distribution of averaged log_2_(S/L) values of the controls via *z*-scores. The generated *z*-scores, one per arm per sample, were assigned to the CNA-affected category if a CNA was detected for at least one bin on the analyzed chromosome arm, and to the CNA-neutral category if this was not the case. Sample sizes per group and per chromosomal arm can be found in Supplementary Data 5.

### Region-set enrichment analysis

For region-set enrichment analysis, only bins that were marked as CNA-neutral were retained. Genomic coordinates of significantly longer and shorter bins compared to the healthy controls were separately used as input to LOLA (v1.1)^54^ and were compared against the universe of coordinates of all CNA-neutral bins for the sample. LOLA hits were deemed significant if their *q*-values were smaller than 0.05. To increase specificity, we summarized LOLA’s output over all EwS samples with genetic tumor evidence and kept only hits that were significant in at least 10 samples. We then sorted the resulting list of hits by either the average genomic tumor content estimation of samples in which an entry was significant (in order to prioritize region sets that are highly tumor-specific) or by the average *q*-value an entry achieved in all EwS samples with genetic tumor evidence (in order to identify the most robustly identified signatures) (Supplementary Data 6).

### LIQUORICE analysis of fragment coverage at regions-of-interest

Genome-wide read coverage was calculated for each cfDNA sample using bamCoverage (deeptools suite v3.1.2)^77^, set to infer the coverage in a fragment-wide manner, to normalize the coverage to 1×, and to require a minimum mapping quality of 20 for a fragment to be counted. To analyze the resulting cfDNA fragment coverage data at predefined regions-of-interest, such as cancer-specific regions of open chromatin, we developed a dedicated method and software, which we called LIQUORICE. Our method takes the characteristic fragment-size distribution of cfDNA into account and analyzes biases at the fragment level. It starts by splitting each region-of-interest into five bins with sizes corresponding to bins of 10%, 15%, 50%, 15%, and 10% of the total length of the region, respectively. This is done in order to facilitate comparisons between regions of different lengths within the same region set. After splitting, every site consists of five bins, regardless of the initial length of the region. Next, the adjacent genomic region (20 kb to both sides) is split into bins of 500 bp. The mean coverage of each bin is then extracted from the pre-calculated BIGWIG files using pyBigWig (v0.3.11; https://github.com/deeptools/pyBigWig) and divided by the coverage value of the corresponding 500 kb window as calculated by ichorCNA^7^ to correct for CNA biases.

Next, a position-weight vector is determined for each bin size, which is used for subsequent bias calculations. The rationale behind this approach is as follows: Because GC bias occurs at the fragment level, usually not only the GC content of a bin itself has an influence on its coverage, but also the GC content of flanking regions. This is the case when there are fragments that overlap the bin and start and/or end outside the bin borders. For an accurate GC bias correction, these flanking regions should not be ignored^79^. To achieve this, we took an approach that is equivalent to sliding fragments of different lengths over a generic bin and determining the positions covered by the fragments as well as the fragments’ influence on the average coverage of the bin (Supplementary Fig. 4a). First, 200 fragment lengths were drawn from the fragment length distribution of the sample. We then assumed that for a given fragment, any starting position relative to the bin start is equally likely. For every fragment length *L* and every starting position *p* in a range of −*L* to (bin size + *L*), the influence on the average coverage of the bin was determined by calculating the fraction of bases in the bin that are overlapped by the fragment starting at *p* and ending at *p* + *L*. The coverage weight of each of the positions covered by the fragment (all positions between *p* and *p* + *L*) was then increased by this influence value. The final coverage weight vector results from summing over all fragment lengths *L* and starting positions *p*. It quantifies the influence on the bin’s coverage for every position relative to the bin start. For a given fragment length distribution, this coverage weight vector is universally applicable to any bin, irrespective of the genomic content.

Once the coverage weight vector has been calculated, the reference DNA sequence is extracted for every genomic bin and its surrounding regions. To determine the GC weight vector, every position (relative to the bin start) is given a weight of zero if its nucleotide is an A or a T, a weight of one for G or C, and a weight of 0.461 (the genome-wide mean of GC content^80^) if the reference is an N. This GC weight vector is then multiplied column-wise with the pre-calculated coverage weight vector. Finally, the resulting vector is summed, and the sum is divided by the sum of the coverage weight vector for normalization to a value between zero and one. The resulting value is the GC bias factor: bins for which the sequence in and around the bin has a high GC content will have a high GC bias factor, while those for which the sequence has a low GC content will have a low bias factor. This value is stored and used for correction with a machine learning algorithm further downstream in the workflow. A similar approach is used for determining bias factors of dinucleotides and trinucleotides. Each dinucleotide and trinucleotide has its own bias factor (except reverse complements, which share a factor). The weight of the corresponding position bias is set to one if the reference sequence starting at the position and extending two (three) bases downstream exactly matches the dinucleotide (trinucleotide), and is zero otherwise.

The calculation of the mappability bias factors is based on mappability tracks calculated with the GEM software^81^ for 75 bp reads, which assign every position in the genome a value representing the mappability of a read of a specified length that starts at that position. We use forward mappability (which we define as the mappability of a 5′ to 3′ read starting at a position *p*), reverse mappability (defined as the mappability of a 3′ to 5′ read starting at *p*, which is equivalent to the forward mappability of *p* minus 75 bp), and the maximum of these two values as variables to estimate the mappability bias. The latter value is included to account for the fact that the mappability of a fragment is determined also by the interaction of the two read mates. For the calculation of mapping bias, different coverage weight vectors than those for GC and di-/trinucleotide bias are required, one each for forward and reverse mappability. For these vectors, only the fragment start (or end, respectively) is assigned the fraction of bases in the bin that are overlapped by the fragment, and all other positions are set to zero.

Next, we trained a random forest with 50 trees using the H2O Python library (http://docs.h2o.ai/h2o/latest-stable/h2o-py/docs/intro.html) on data from all regions in a given region set, with coverage as the response variable and the bias factors as predictors. The five central bins that cover the core region are excluded from the training. The trained model is then used to predict coverage of each bin, based on its bias factors. To obtain corrected coverage information, the resulting values are subtracted from the uncorrected coverage values (Supplementary Fig. 4b). After having obtained bias-corrected coverage values, these values are aggregated across all regions in the region set using the mean, resulting in a single coverage profile.

In the next step, these coverage profiles are quantified using a model-based fitting approach tailored to the biological aspects of nucleosome occupancy at gene-regulatory regions, which may be regulated at three levels: (i) Transcription factor binding sites; (ii) enhancer or promoter regions; and (iii) large co-regulated genomic segments such as super-enhancers. To account for these three levels of regulation, we fitted three Gaussian functions of different widths as well as an intercept to the aggregated, bias-corrected coverage profile. These functions were constrained to be centered in the middle of the regions-of-interest. Moreover, their *σ* parameters, which determine the widths of these functions, were constrained to rough estimates of the genomic widths of the biological signals that they represent: 20–200 bp for transcription factor binding sites, 200–3000 bp for enhancer or promoter regions, and 3000–40,000 bp for super-enhancers. *σ* values and amplitudes were then optimized with the Python package lmfit (10.5281/zenodo.1469545) using dampened least-square-optimization^82^. After the optimization was performed for every sample, the sample-wise medians of the three *σ* values were obtained and used as fixed constraints for an additional optimization run. After the second fitting process, the following parameters were used to quantify the dip strength and shape: The area over the curve (AOC) between the intercept and the fitted combined model, the heights of the three Gaussian functions relative to zero, the intercept value, and the total dip depth (Supplementary Fig. 4c and Supplementary Data 9).

### Machine learning model for tumor detection and classification

For tumor detection and classification, we used four alternative machine learning algorithms: Linear support vector machines (which tend to perform well even on small data sets), feed-forward neural networks (which provide high flexibility), random forests (which tend to perform well without any parameter optimization), and binomial generalized linear models with elastic-net regularization (which provide a relatively straightforward baseline method). These algorithms were trained and evaluated using the following bootstrapping and cross-validation scheme: In 100 iterations, *n* patients were drawn with replacement from the full data set of *n* patients. Samples of drawn patients were assigned to the training set, while all other samples were assigned to the test set. Iterations with <5 samples of each class in the test set were rejected and repeated. In each iteration, the training set was split once more using stratified 5-fold cross-validation, and inner cross-validation scores were used to select the algorithm and hyperparameters. The best algorithm/hyperparameter combination was selected from the following options: (i) Linear support vector machines (hyperparameter C: grid search over [2^−5^, 2^−3^, 2^−1^, 2^1^, 2^3^, 2^7^, 2^9^, 2^11^, 2^13^, 2^15^]; as implemented in scikit-learn^83^); (ii) feed-forward neural networks (using a rectifier activation function, adaptive learning rate, and two hidden layers of size 200 each; as implemented in H2O’s Python API); (iii) random forests (with 200 trees; as implemented in H2O’s Python API); (iv) binomial generalized linear models with elastic-net regularization (hyperparameter alpha: grid search over [0.1, 0.5, 0.7, 0.9, 0.95, 0.99, 1]; activated lambda search; as implemented in the H2O Python library). For the latter three, the minority class was set to be oversampled by a factor calculated as the ratio between the number of samples in the majority class divided by the number of samples in the minority class. After the best model was selected, its predictions on the (unseen) test set were stored for each of the 100 iterations. To obtain the performance evaluation of the classifier, a ROC curve was calculated for each iteration, and an aggregated ROC curve and its AUC value were calculated by averaging over the 100 individual curves.

In addition to the individual classifiers, a meta-learner was designed as follows: In each of the 100 bootstrap iterations, the predicted tumor probabilities were recorded for each of the four trained prediction models (using one model each for global fragment size, regional fragmentation, read depth, or coverage at EwS-specific DHSs, selected based on the performance in the inner cross-validation). The meta-learner used the four resulting predictions per sample as input features, combining and weighing the information from different fragment-based metrics. To avoid data leakage between training and test sets, we made sure that only samples in the training set of a given iteration were used to derive the input features used for training of the meta-learner in the same iteration. The meta-learner consisted of a Gaussian generalized linear model, as described above. Again, grid search was performed using the training data only.

The following feature sets were used as input for the machine learning algorithms: (i) Global fragment size: *P*(100–150), *P*(160–180), *P*(180–220), *P*(250–320), *P*(100–150)/P(163–169), *P*(160–180)/*P*(180–220), the amplitude at 10 bp. Here, *P*(*x* − *y*) stands for the proportion of reads in a size range from *x* to *y* bp. Moreover, the amplitude at 10 bp was based on the local minima at 84, 96, 106, 116, 126, 137, and 148 bp and the local maxima at 81, 92, 102, 112, 122, 134, 144 bp. This set of features was chosen in concordance to those reported by Mouliere et al.^49^, although we excluded features utilizing the range of fragments 20–100 bp, as we detected minor technical artifacts in some of our samples around 20 bp. (ii) Coverage drop around EwS-specific DHSs: Total dip area based on the combination of the three fitted Gaussian functions G1 (narrowest) to G3 (widest) and the fitted intercept, total dip area excluding the range [−*σ*_G1_,*σ*_G1_], sum of *y* values of the fitted model over all bins, amplitudes of G1, G2, and G3, the intercept, and the total dip depth (i.e., the sum of heights of G1, G2, and G3). We added the total dip area excluding the range [−*σ*_G1_, *σ*_G1_] as a metric that assesses the signal independent of the change directly at the center of each DHS, and the sum of *y* values of the fitted model over all bins as a metric that combines intercept and dip area. (iii) Read-depth in 5 Mb bins: The number of fragments in the size range 100–220 bp in 380 bins with a size of 5 Mb each, GC-corrected using LOESS smoothing (separately for fragments sized 100–150 bp and 151 to 220 bp), and *z*-transformed within each sample. The bin size of 5 Mb was chosen to allow good comparability to regional fragmentation-based classifiers. (iv) Regional fragment size: A combination of read depth in 5 Mb bins, regional read depth of short fragments in 5 Mb bins, corresponding to the number of fragments in the size range 100–150 bp in 380 bins with a size of 5 Mb each, GC-corrected using LOESS smoothing and *z*-transformed within each sample, and read depth in chromosomal arms, corresponding to the regional read depth in 5 Mb bins (before *z*-transformation), averaged over all bins in the same chromosomal arm, and then normalized by the sum over all arms within each sample. This set of features was chosen in reference to the features used by Cristiano et al.^32^.

To increase the number of healthy controls in the machine learning analysis, in addition to the 22 healthy individuals in our data set we also included published whole-genome sequencing data for 46 healthy individuals from two independent data sets (Ulz et al.^34^, *n* = 24; Christiano et al.^32^, *n* = 22; Supplementary Data 10). These samples were sequenced with comparable coverage as the cfDNA samples included in this study (Cristiano et al.: 10×, Ulz et al.: 23×). To remove systematic differences between the three data sources for healthy individuals (this study, Cristiano et al., Ulz et al.), for each data set and each feature in the data set, the mean and standard deviation over the healthy control samples were determined, and all entries in the data set were normalized by subtracting the mean and dividing by the standard deviation. As the result, the three sets of healthy controls had the same mean and standard deviation, and they could all be used as references for the cancer samples in our data set. As a measure against information leakage across data sets, we employed a meta-analysis approach, and trained separate machine learning models for distinguishing EwS samples from each of the three sets of healthy controls. We then integrated the information across data sets by taking the mean of the ROC data obtained from the 3*100 bootstrap iterations as our final performance estimate. Independently of that approach, we also investigated the performances of machine learning classifiers using only one of the three control sets and found that the results were similar between the sets (Supplementary Fig. 6b). We also tested the efficiency of this normalization by training machine learning classifiers (as described above) to distinguish between healthy controls generated in this study and, separately, healthy controls from the other two studies. Since none of the classifiers achieved better-than-random prediction performance, we concluded that the normalization was successful. Of note, the performance of these classifiers was worse than expected by chance, with ROC AUC values well below 0.5 (Supplementary Fig. 6a), which could be explained as follows: Because the normalization was performed on the complete data set (prior to splitting into train and test sets), in every iteration there were minor random differences in the distributions of feature values in the training set between the two classes (our data set versus the other data set). The machine learning classifiers pick up these differences during training. However, since by definition there are no differences in the mean between the two classes overall (i.e., in the full data set), the samples in the test set will show opposite between-class differences than those in the test set, resulting in systematically wrong predictions on test set samples and ROC AUC values below 0.5. Following this explanation, one would expect that the higher the number of features in a set, the more extreme some of the randomly observed between-class differences can become. Indeed, we found that read depth and regional fragmentation, for which this applies, performed worst.

### Associations with clinical data

Kaplan–Meier plots and statistics, as well as Cox proportional hazards models, were generated using the survival package in R (v3.1-12). For relapse-free survival from time of diagnosis (RFS), we used Cox proportional hazards models for a multivariate analysis that corrected for sex and gender. These Cox models could not be applied for overall survival (OS), as none of the patients without detected tumor-derived cfDNA died in the observed period. However, we found that age and sex were not significantly associated with OS (*p* = 0.77 and 0.88, log-rank test). To correct for the percentage of tumor-derived cfDNA (based on genetic methods) and for technical (number of PCR cycles, amount of input DNA) and biological (sex, age) covariates as shown in Supplementary Fig. 10, we built a linear multivariate regression model that used these covariates to predict each fragment-based metric, and used the residuals for further analysis.

### Reporting summary

Further information on research design is available in the Nature Research Reporting Summary linked to this article.

## Supplementary information

Supplementary Information

Description of Additional Supplementary Files

Supplementary Data 1

Supplementary Data 2

Supplementary Data 3

Supplementary Data 4

Supplementary Dataset 5

Supplementary Dataset 6

Supplementary Dataset 7

Supplementary Dataset 8

Supplementary Dataset 9

Supplementary Dataset 10

Reporting Summary

## Data Availability

The sequence data have been deposited at the European Genome-phenome Archive (EGA), which is hosted by the EBI and the CRG, under accession number EGAS00001005127. This data is available under a controlled access regimen to ensure the protection of personally identifiable data; access can be obtained by contacting E.M.T. Publically available sequencing data for cfDNA from healthy individuals were accessed via the EGA (EGAD00001005343, and EGAD00001005339). Pre-processed, de-identified data are available as an open-access online resource for viewing and download from the following website: http://ews-liquid-biopsy.computational-epigenetics.org. The remaining data are available within the Article, Supplementary Information, or available from the authors upon request.

## Code availability

The analysis source code underlying the final version of the paper is provided under open access on the following website: http://ews-liquid-biopsy.computational-epigenetics.org. A code archive is also available on Zenodo.
