# Supplementary material for: Multimodal analysis of cell-free DNA whole-genome sequencing for pediatric cancers with low mutational burden
Source: Nat Commun. 2021 May 28;12:3230. doi: 10.1038/s41467-021-23445-w (PMC8163828; doi:10.1038/s41467-021-23445-w)
Supplement: Supplementary file 1 — Supplementary Information [file 41467_2021_23445_MOESM1_ESM.pdf]

Supplementary Figures

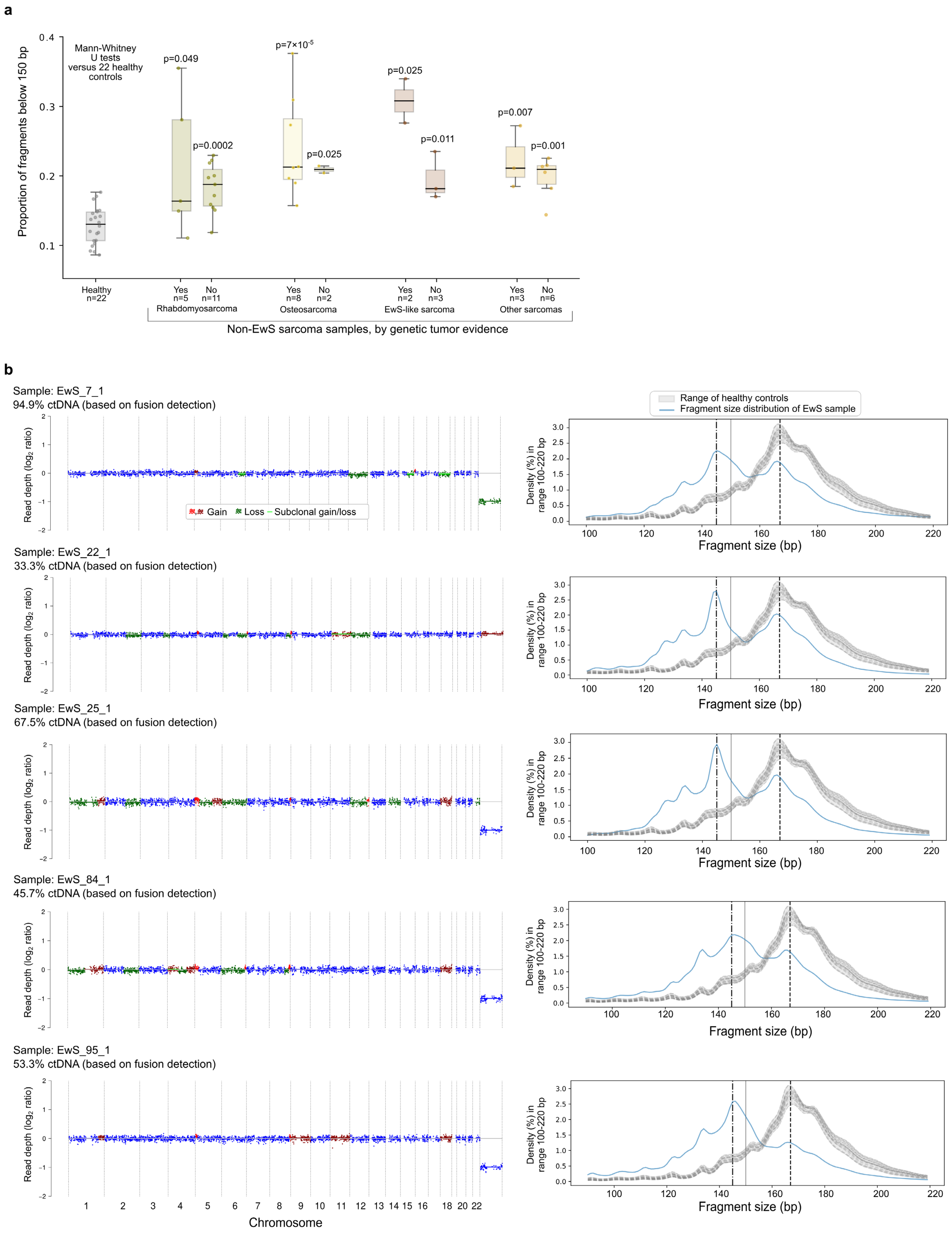

Supplementary Figure 1. *Additional analyses of the global fragment size distribution in cfDNA samples from patients with pediatric sarcomas.* (a) Proportion of short cfDNA fragments (20-150 bp) for non-EwS sarcomas and healthy controls. Boxes correspond to interquartile ranges (IQR), black lines indicate the median, and the whiskers extend to the lowest or highest data points that are still within 1.5 IQR of the bottom or top quartile, respectively. Significance versus the 22 healthy controls was assessed using two-sided Mann-Whitney U test.

Yes/No indicate the presence of ctDNA based on genetic evidence (**Supplementary Data 2**). (b) Global cfDNA fragmentation in patients with no detectable CNAs. CNA plots (left, ichorCNA) and density plots for global fragmentation patterns (right) are shown for five samples (EwS_7_1, EwS_22_1, EwS_25_1, EwS_84_1, EwS_95_1). These five samples have high ctDNA content (>30%) but no clearly detectable CNAs at 12x WGS coverage.

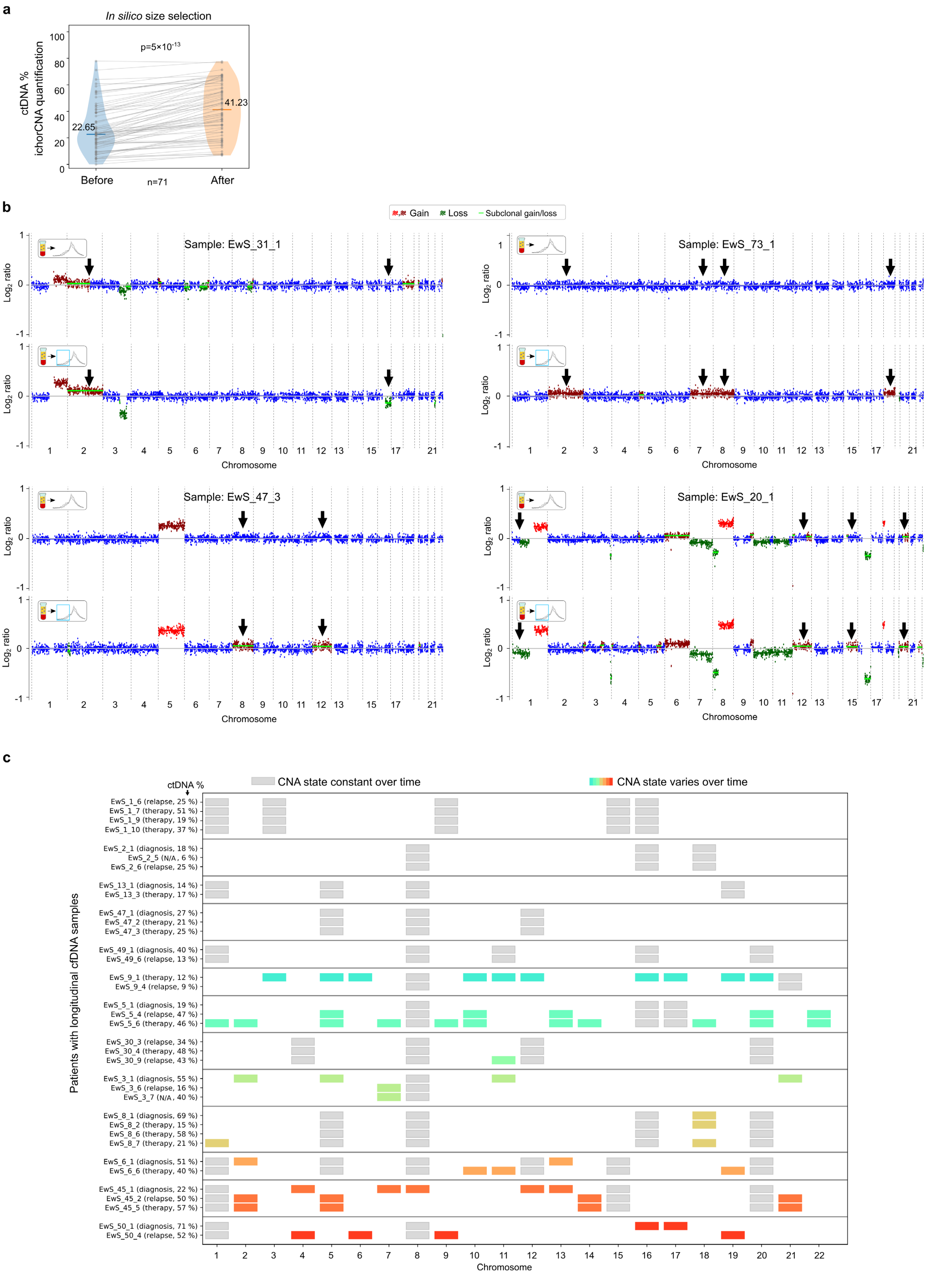

Supplementary Figure 2. *Impact of in silico size selection on CNA detection and tumor evolution monitoring over time.* (a) Estimated proportion of tumor-derived DNA based on CNAs using ichorCNA[1], calculated before (left) and after (right) in silico size selection for short DNA fragments (90-150 bp). Lines connect the same samples; blue and orange lines indicate the median of the two distributions. Significance was assessed using

the Wilcoxon signed-rank test (two-sided, n=71 samples analyzed both before and after size-selection). (b) CNA plots (ichorCNA) of four EwS cfDNA samples (EwS_31_1, EwS_73_1, EwS_47_3, EwS_20_1) before (top) and after (bottom) in silico size selection, which enhanced the discovery of (sub-clonal) CNAs (indicated by black arrows). Inferred amplifications are shown in red, inferred deletions are shown in green, and CNA-neutral regions are shown in blue. (c) CNA profiles in patients with longitudinal samples with at least 5% ctDNA based on ichorCNA (n=13 patients). Gray color indicates CNAs that remain constant during disease progression; other colors (one per patient) represent CNAs that change over time.

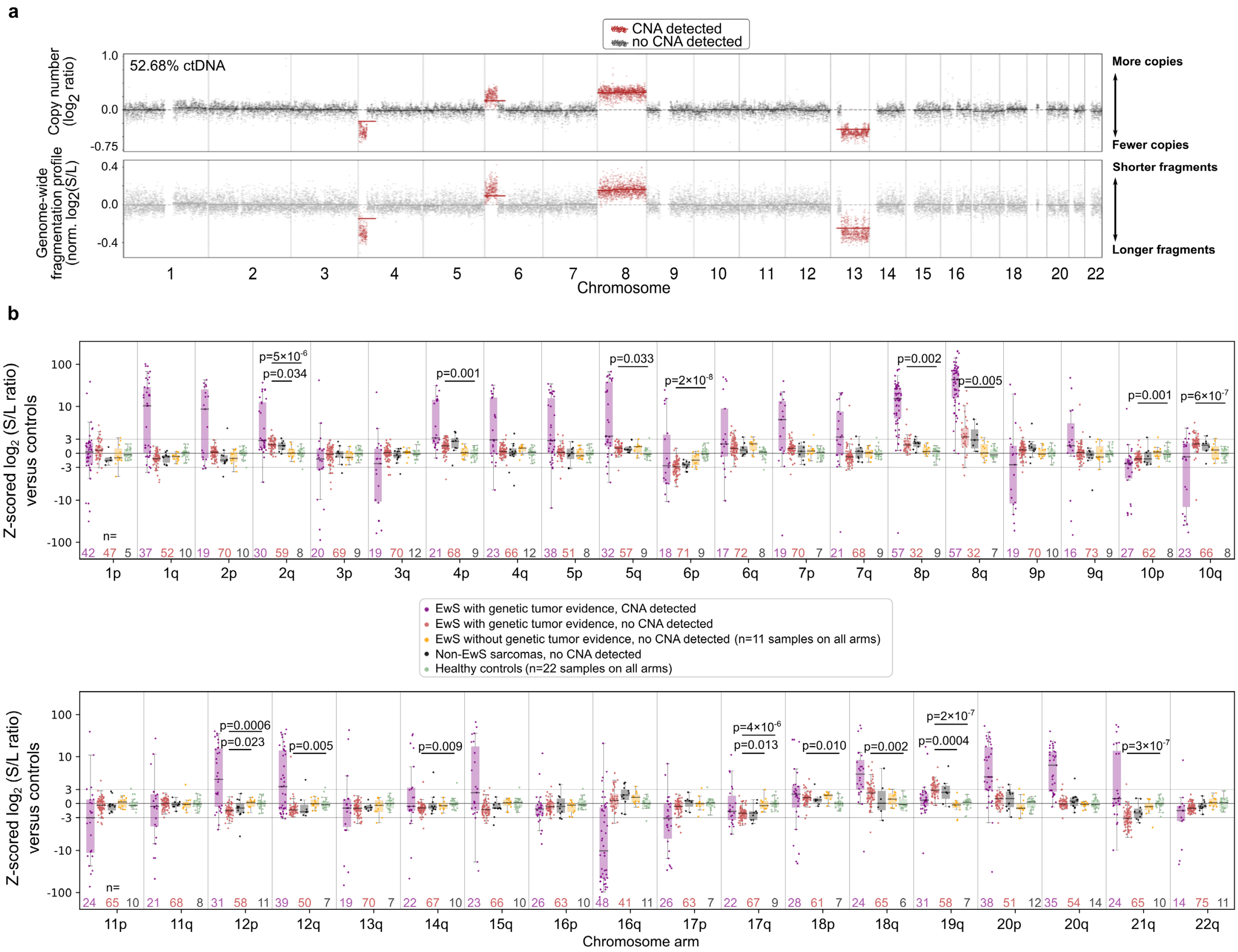

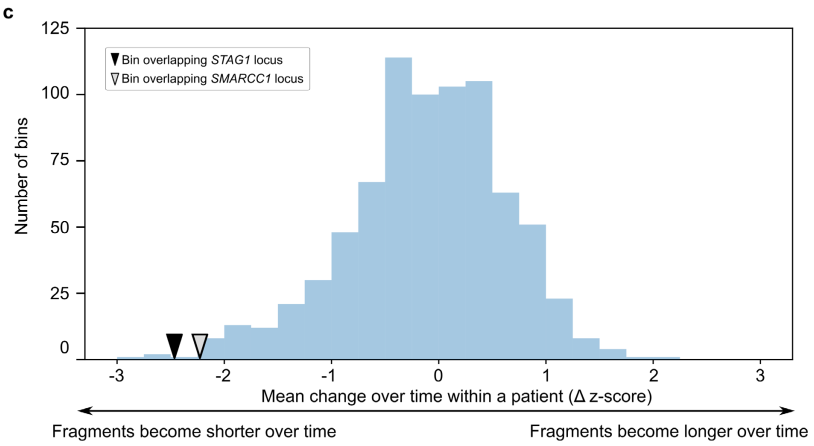

d

Chr.	*Start*	*End*	*Overlapping genes*
chr3	136839100	136939100	SLC35G2, NCK1-DT,NCK1
chr1	64900000	65000000	JAK1, LINC01359
chr9	14900000	15000000	FREM1, LOC389705
chr1	28900000	29000000	EPB41
chr3	136539100	136639100	STAG1
chr3	47600000	47700000	SMARCC1, SNORD146
chr9	116100000	116200000	PAPPA
chr3	58228500	58328500	ABHD6, HTD2,RPP14
chr3	170100000	170200000	PHC3
chr9	20600000	20700000	MLLT3,FOCAD,FOCAD-AS1

Supplementary Figure 3. *Additional analyses relevant to regional cfDNA fragmentation patterns.* (a) CNA plots (top, based on ichorCNA) and genome-wide fragmentation profile (normalized log_2(S/L ratio) per bin; bottom), in a cfDNA sample with high ctDNA content (EwSLike_1_3; 53%) and highly enriched in short DNA fragments. Note that bins in regions affected by copy number gains are enriched for ctDNA and therefore have shorter-than-average cfDNA fragments in this highly fragmented cfDNA sample, whereas bins in regions affected by copy number losses are depleted for ctDNA and therefore have fragments longer than the genome-wide average of the sample. (b) Boxplots showing regional fragmentation at the chromosomal arm level in cfDNA samples from patients with EwS and other pediatric sarcomas compared to healthy controls. EwS cfDNA samples are grouped by whether they are CNA-affected (purple) or CNA-neutral at the corresponding chromosome arm as well as by genetic-based evidence for ctDNA detection (red and yellow). For non-EwS only samples with genetic-based ctDNA evidence and without detected CNAs on the chromosomal arm are

shown (black). Healthy controls are shown in green. CNA-neutral chromosome arms with significantly different fragment lengths in EwS samples with genetic tumor evidence, compared to (i) non-EwS sarcomas with genetic tumor evidence, (ii) healthy controls, (iii) EwS without genetic tumor evidence are indicated (two-sided Mann-Whitney U test; Bonferroni-corrected p-values are shown.). Boxes correspond to interquartile ranges (IQR), black lines indicate the median, and the whiskers extend to the lowest or highest data points that are still within 1.5 IQR of the bottom or top quartile, respectively. (c) Histogram showing the average change over time of each bin's z-scored $\log_2$(S/L) ratio across all seven patients included in this analysis. Only bins overlapping with EwS-specific DHSs were analyzed. Also, only bins which were CNA-neutral in at least six time point pairs are included, in order to limit variability due to random fluctuations. Two of the most consistently changing genomic bins (towards shorter fragments over time; ranked fifth and sixth overall) were located within the encoding regions for genes *STAG1* and *SMARCC1* (*BAF155*) and are marked with black and gray triangles, respectively. (d) Table listing the ten most consistently changing genomic bins towards shorter fragments over time. Genomic coordinates (hg38) of the bins and the genes encoded within the corresponding regions are provided.

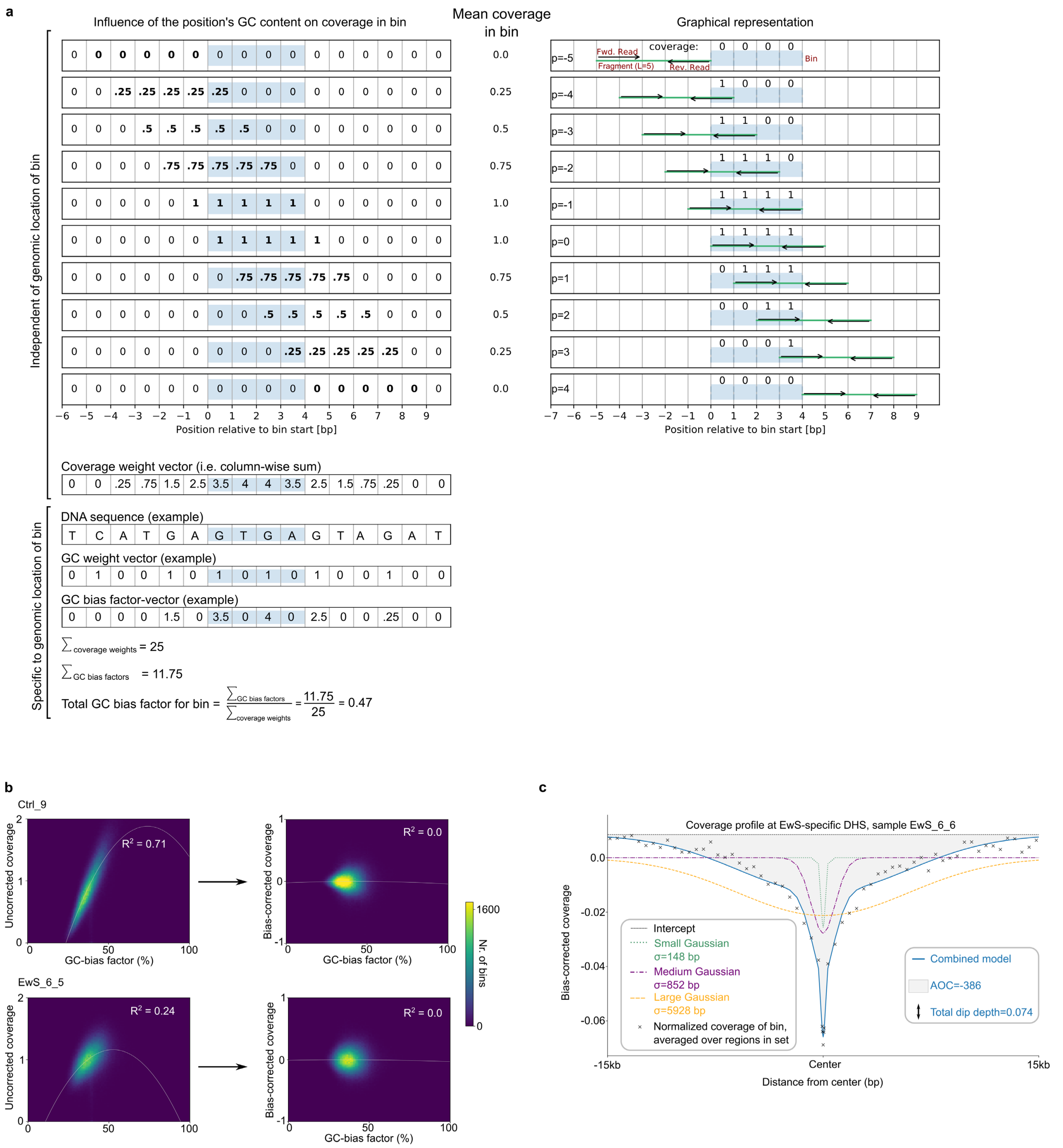

Supplementary Figure 4. *Illustration of the bias-correction and coverage quantification by LIQUORICE.* (a) Diagram illustrating the fragment-sliding method used for determination of GC-content biases in LIQUORICE. The coverage weight vector is averaged from the results of many different fragment lengths, drawn from the sample's global fragment size distribution (see Methods). *p*: starting position of fragment relative to bin start; *L*: fragment length. (b) Correlation between the GC content (GC bias factor) and coverage signal at EwS-specific DHSs before (left) and after (right) correction. Coverage signal is shown for a healthy control (top) and a EwS (bottom) cfDNA sample. R^2 values are with respect to the fitted quadratic function shown in white, chosen to recapitulate the unimodal nature of GC bias[2]. (c) GC-bias corrected coverage signal at EwS-specific DHSs in sample EwS-6_6, an example of the model-based fitting approach used for quantification of the coverage signal. Three Gaussian functions of different widths (σ values) are combined for quantification of the coverage signal accumulated from all regions in the region-set of interest. Key output parameters include the total dip depth and the area over the curve (AOC).

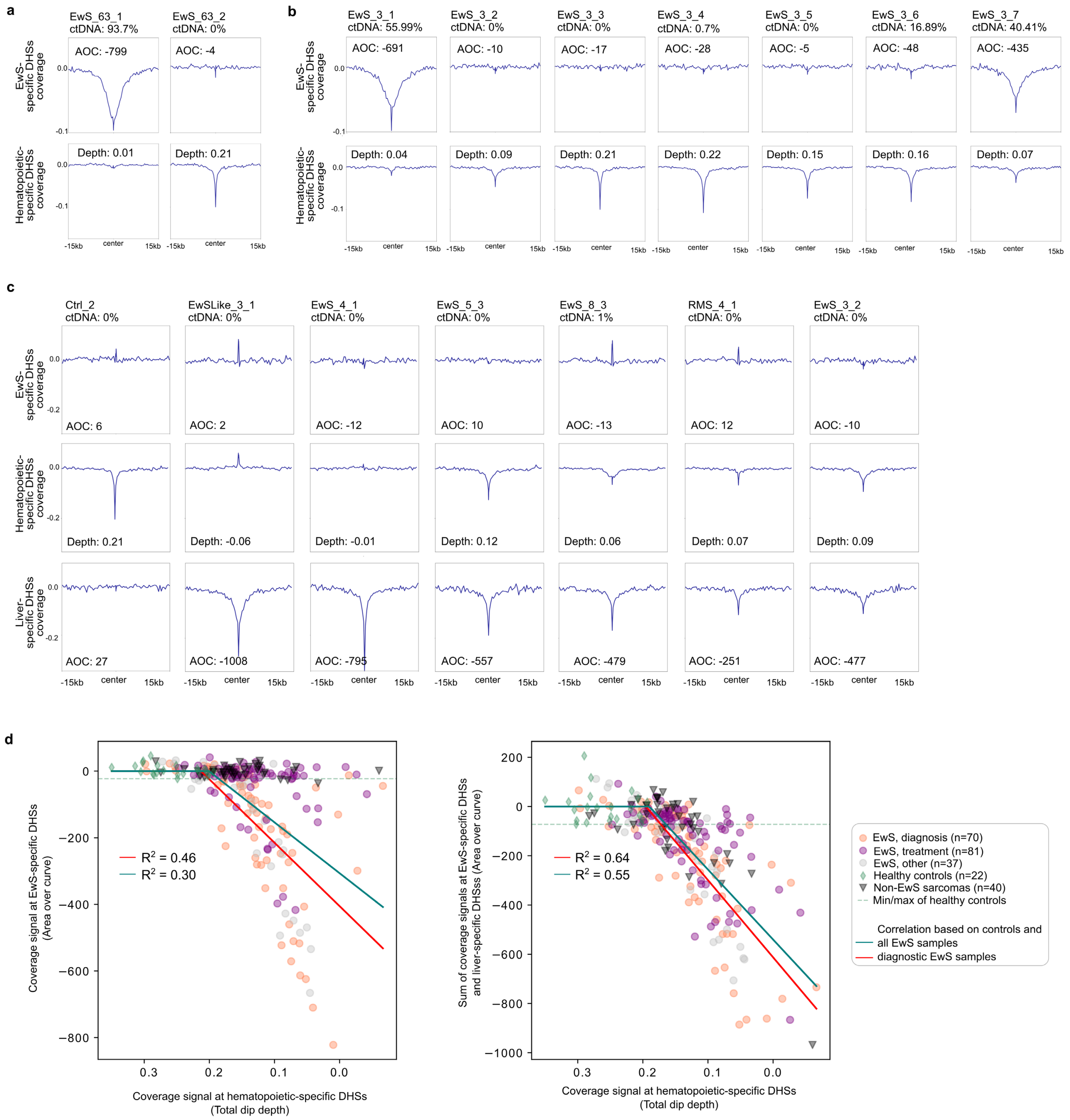

Supplementary Figure 5. *Coverage signal at EwS-specific, hematopoietic, and liver-specific DHSs.* (a-b) Plots illustrating the anticorrelation between the coverage signals at EwS-specific DHSs and the hematopoietic-specific DHSs. Longitudinal samples from two EwS patients are shown: EwS_63 (panel a) and EwS_3 (panel b). (c) Plots illustrating the impact of liver damage on the coverage signal of hematopoietic-specific DHSs. Shown are samples from a healthy control and patients with EwS-like sarcoma (EwSLike_3_1), EwS (EwS_4_1, EwS_5_3, EwS_8_3, EwS_3_2), and rhabdomyosarcoma (RMS_4_1), which had no detectable tumor-derived DNA at the time of sample collection. The genetically inferred tumor-derived DNA content as well as the AOC (EwS-specific and liver-specific DHSs) and total dip depth (hematopoietic-specific DHSs) are shown for each sample. (d) Anticorrelation between the coverage signals at hematopoietic-specific DHSs and the coverage signal at EwS-specific DHSs alone (left) or the sum of the coverage signals at EwS-specific and liver-specific DHSs (right).

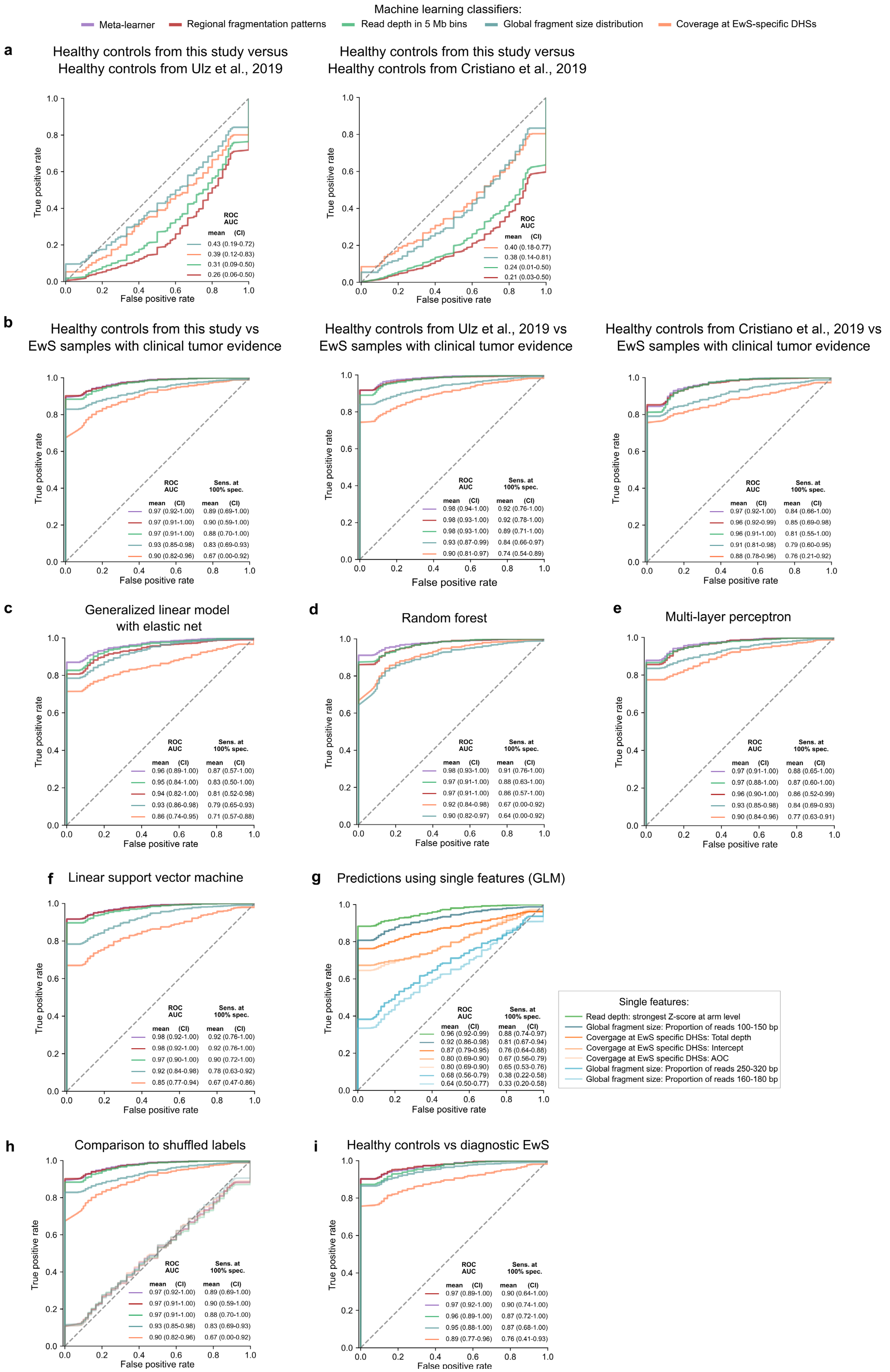

Machine learning classifiers:
Meta-learner
Regional fragmentation patterns
Read depth in 5 Mb bins
Global fragment size distribution
Coverage at EwS-specific DHSs
a
Healthy controls from this study versus Healthy controls from Ulz et al., 2019
ROC AUC mean (CI)
0.43 (0.19-0.72)
0.39 (0.12-0.83)
0.31 (0.09-0.50)
0.26 (0.06-0.50)
Healthy controls from this study versus Healthy controls from Cristiano et al., 2019
ROC AUC mean (CI)
0.40 (0.18-0.77)
0.38 (0.14-0.81)
0.24 (0.01-0.50)
0.21 (0.03-0.50)
True positive rate
False positive rate
b
Healthy controls from this study vs EwS samples with clinical tumor evidence
ROC AUC mean (CI) | Sens. at 100% spec. mean (CI)
0.97 (0.92-1.00) 0.89 (0.69-1.00)
0.97 (0.91-1.00) 0.90 (0.59-1.00)
0.97 (0.91-1.00) 0.88 (0.70-1.00)
0.93 (0.85-0.98) 0.83 (0.69-0.93)
0.90 (0.82-0.96) 0.67 (0.00-0.92)
Healthy controls from Ulz et al., 2019 vs EwS samples with clinical tumor evidence
0.98 (0.94-1.00) 0.92 (0.76-1.00)
0.98 (0.93-1.00) 0.92 (0.78-1.00)
0.98 (0.93-1.00) 0.89 (0.71-1.00)
0.93 (0.87-0.99) 0.84 (0.66-0.97)
0.90 (0.81-0.97) 0.74 (0.54-0.89)
Healthy controls from Cristiano et al., 2019 vs EwS samples with clinical tumor evidence
0.97 (0.92-1.00) 0.84 (0.66-1.00)
0.96 (0.92-0.99) 0.85 (0.69-0.98)
0.96 (0.91-1.00) 0.81 (0.55-1.00)
0.91 (0.81-0.98) 0.79 (0.60-0.95)
0.88 (0.78-0.96) 0.76 (0.21-0.92)
c
Generalized linear model with elastic net
0.96 (0.89-1.00) 0.87 (0.57-1.00)
0.95 (0.84-1.00) 0.83 (0.50-1.00)
0.94 (0.82-1.00) 0.81 (0.52-0.98)
0.93 (0.86-0.98) 0.79 (0.65-0.93)
0.86 (0.74-0.95) 0.71 (0.57-0.88)
d
Random forest
0.98 (0.93-1.00) 0.91 (0.76-1.00)
0.97 (0.91-1.00) 0.88 (0.63-1.00)
0.97 (0.91-1.00) 0.86 (0.57-1.00)
0.92 (0.84-0.98) 0.67 (0.00-0.92)
0.90 (0.82-0.97) 0.64 (0.00-0.92)
e
Multi-layer perceptron
0.97 (0.91-1.00) 0.88 (0.65-1.00)
0.97 (0.88-1.00) 0.87 (0.60-1.00)
0.96 (0.90-1.00) 0.86 (0.52-0.99)
0.93 (0.85-0.98) 0.84 (0.69-0.93)
0.90 (0.84-0.96) 0.77 (0.63-0.91)
f
Linear support vector machine
0.98 (0.92-1.00) 0.92 (0.76-1.00)
0.98 (0.92-1.00) 0.92 (0.76-1.00)
0.97 (0.90-1.00) 0.90 (0.72-1.00)
0.92 (0.84-0.98) 0.78 (0.63-0.92)
0.85 (0.77-0.94) 0.67 (0.47-0.86)
g
Predictions using single features (GLM)
0.96 (0.92-0.99) 0.88 (0.74-0.97)
0.92 (0.86-0.98) 0.81 (0.67-0.94)
0.87 (0.79-0.95) 0.76 (0.64-0.88)
0.80 (0.69-0.90) 0.67 (0.56-0.79)
0.80 (0.69-0.90) 0.65 (0.53-0.76)
0.68 (0.56-0.79) 0.38 (0.22-0.58)
0.64 (0.50-0.77) 0.33 (0.20-0.58)
Single features:
Read depth: strongest Z-score at arm level
Global fragment size: Proportion of reads 100-150 bp
Covergage at EwS specific DHSs: Total depth
Covergage at EwS specific DHSs: Intercept
Covergage at EwS specific DHSs: AOC
Global fragment size: Proportion of reads 250-320 bp
Global fragment size: Proportion of reads 160-180 bp
h
Comparison to shuffled labels
0.97 (0.92-1.00) 0.89 (0.69-1.00)
0.97 (0.91-1.00) 0.90 (0.59-1.00)
0.97 (0.91-1.00) 0.88 (0.70-1.00)
0.93 (0.85-0.98) 0.83 (0.69-0.93)
0.90 (0.82-0.96) 0.67 (0.00-0.92)
i
Healthy controls vs diagnostic EwS
0.97 (0.89-1.00) 0.90 (0.64-1.00)
0.97 (0.92-1.00) 0.90 (0.74-1.00)
0.96 (0.89-1.00) 0.87 (0.72-1.00)
0.95 (0.88-1.00) 0.87 (0.68-1.00)
0.89 (0.77-0.96) 0.76 (0.41-0.93)

Supplementary Figure 6. *Evaluation of the batch correction procedure and further classification results.* ROC curves as well as AUC and sensitivity values were averaged over 100 bootstrap iterations. CI is the 95% confidence interval obtained from bootstrapping. (a) After batch correction, machine learning classifiers are unable to distinguish healthy controls generated in this study (n=22) from those obtained from Cristiano et al.[3] (left; n=22) and Ulz et al.[4] (right, n=24), respectively. The poorer performance than expected by chance could be explained by the train/test splits (see Methods). (b) Performance of classifiers trained to distinguish cfDNA samples from patients with EwS and clinical tumor evidence ($n_{samples}$=103) from healthy controls from this study (left), Cristiano et al[3] (middle), and Ulz et al[4] (right). The performance of each of the classifiers at 12x coverage is shown. (c-f) ROC curves showing the influence of the machine learning algorithm on classification performance. All ROC curves refer to the task of distinguishing EwS cfDNA samples with clinical tumor evidence (n=103) from healthy controls generated in this study (n=22). The performance of the classifiers when the machine learning algorithm is fixed (instead of selected by inner cross-validation as in Fig. 6) is shown. The following algorithms were used: generalized linear model with elastic net regularization (c); random forest (d); multi-layer perceptron (e); linear support vector machine (f). (g) Performance of single features when set to distinguish EwS samples with clinical tumor evidence (n=103) from healthy controls generated in this study (n=22). For this task, general linear models were used (as described in Methods). (h) Comparison of the performance of the classifiers with the correct class labels (strong colors) vs the performance when the class labels were randomized (averaged over 30 iterations of label shuffling, each evaluated by 10*bootstrapping (faint colors) when set to distinguish EwS samples with clinical tumor evidence (n=103) from healthy controls generated in this study (n=22). (i) Performance of classifiers for distinguishing diagnostic EwS samples with clinical tumor evidence (n=64) from healthy controls generated in this study (n=22).

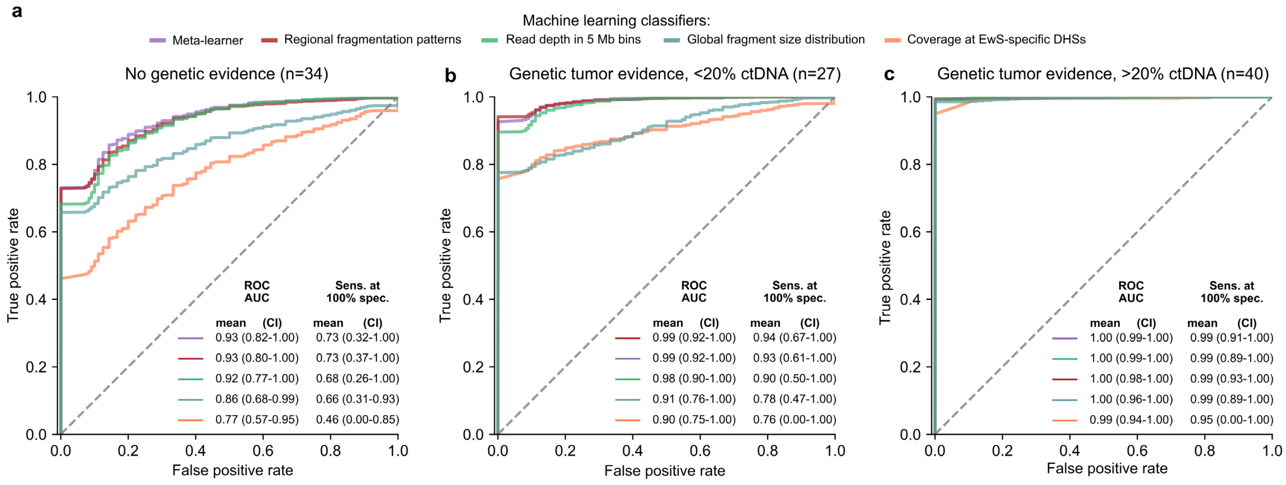

d

Feature-set	Percent of detected EwS samples (at 100% specificity) Missing values (N/A) included in the analysis; n=103	Percent of detected EwS samples (at 100% specificity) Missing values (N/A) excluded from analysis; n=56
WGS breakpoint detection	**48%** (49 samples detected, 35 N/A, 19 negative)	**68%** (38 samples detected, 18 negative)
ddPCR	**44%** (45 samples detected, 47 N/A, 12 negative)	**79%** (44 samples detected, 12 negative)
ichor CNA (hand-curated)	**52%** (54 samples detected, 49 negative)	**59%** (33 samples detected, 23 negative)
Combined genetic evidence	**65%** (67 samples detected, 2 N/A, 34 negative)	**79%** (44 samples detected, 12 negative)
Machine learning: Meta-learner, 12x	**84%**	**90%**
Machine learning: Meta-learner, 1x	**86%**	**90%**
Machine learning: Meta-learner, 0.1x	**76%**	**83%**

Supplementary Figure 7. *Comparison of ctDNA detection by genetic-based methods and fragment-based machine learning classifiers*. (a-c) Performance of the classifiers from Fig. 6 (~12x coverage, averaged over 3*100 iterations of bootstrapping) when only sub-groups of EwS cfDNA samples with clinical tumor evidence are used in the test-set: (a) only samples for which no ctDNA could be detected by genetic methods; (b) only samples with detected ctDNA based on genetic evidence, with ctDNA levels below 20%; (c) only samples with more than 20% detected ctDNA based on genetic evidence. (d) Comparisons of the sensitivities between genetic-based methods (based on 12x sequencing coverage) and machine learning (meta-learner) at different coverages. In order to compare machine learning classifiers to genetic-based methods, we calculated their sensitivity at 100% specificity as the percentage of EwS cfDNA samples in the test-set with higher predicted tumor probability than all healthy controls in the test-set, averaged over 100 iterations of bootstrapping. In order to obtain a more conservative estimate of this sensitivity, we calculated this number separately for each of the three sets of healthy controls and took the minimum of these three numbers as the final estimate shown in the table. The middle column reflects the most common scenario, in which some genetic-based measurements cannot be performed (e.g. lacking breakpoint information or repetitive genetic regions preventing ddPCR design). As one cannot detect tumor if no measurement can be performed, and missing values reflect limitations of the genetic methods, here, we count these cases as not detected (i.e. negative). The last column focuses on the subset of samples for which measurements could be performed with all four genetic methods and represents an ideal scenario for genetic approaches.

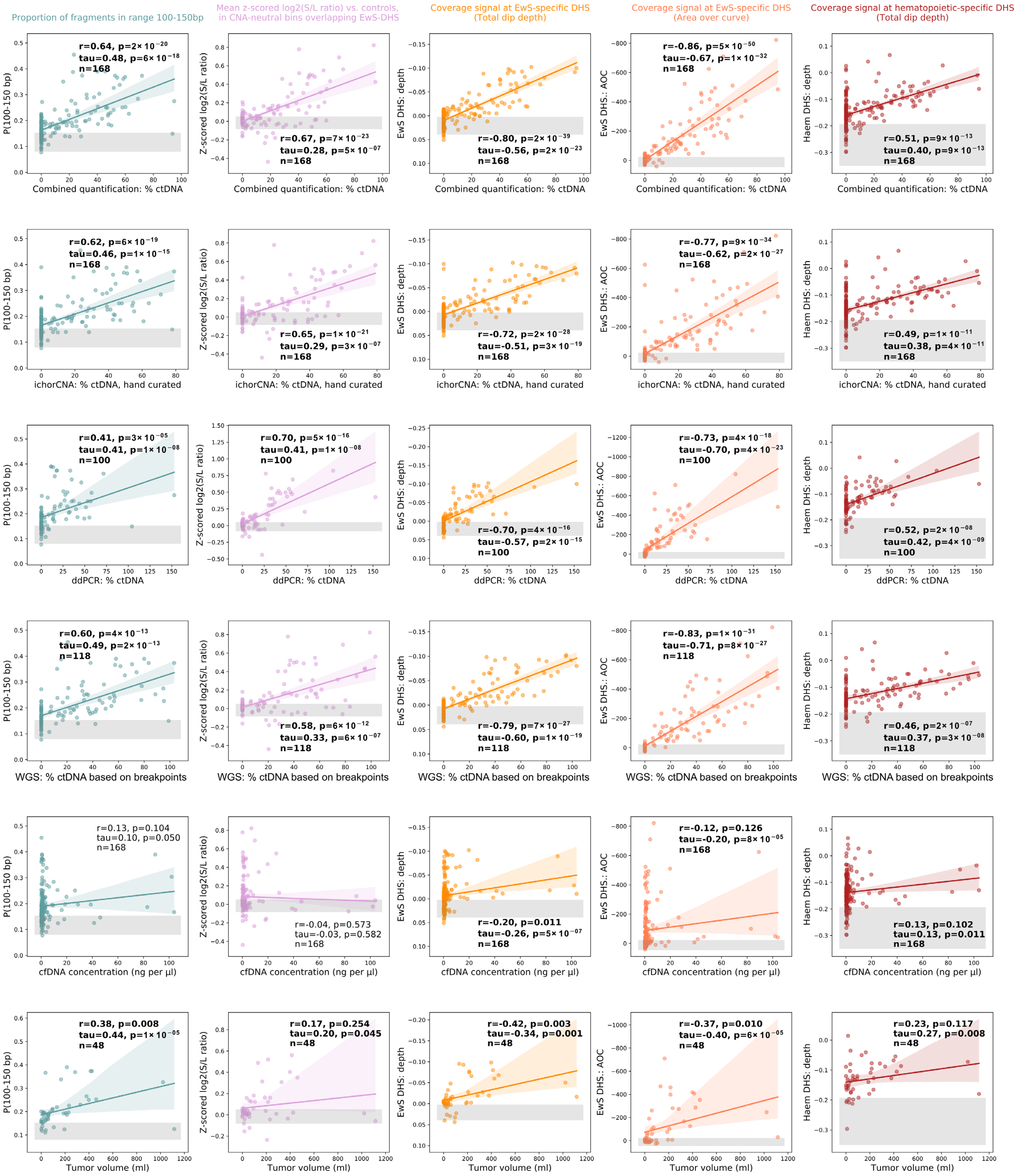

Supplementary Figure 8. *Correlations between several fragment-based metrics and genetic-based ctDNA quantification measures, cfDNA concentration, and tumor volume for cfDNA samples from patients with EwS.* Lines represent linear regression models fitted on the data; colored shaded areas represent the 95% confidence interval of the regression (estimated by bootstrapping); gray shaded areas represent the range of the 22 healthy controls from this study. Bold font indicates significant correlations. p-values (two-sided; without corrections for multiple testing) were calculated using SciPy's stats.pearsonr and stats.kendalltau functions.

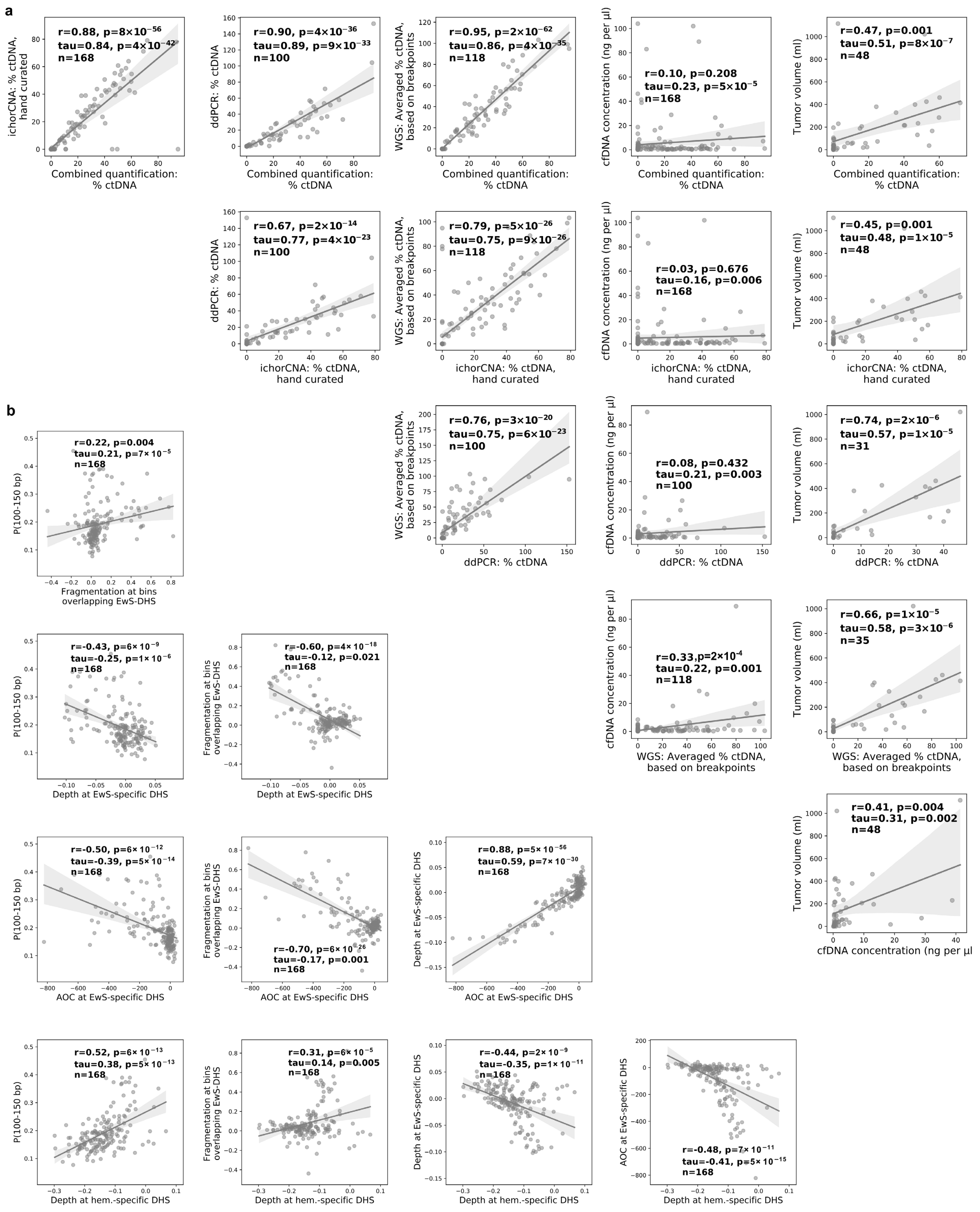

Supplementary Figure 9. *Correlations between (a) individual genetic-based ctDNA quantification measures and to cfDNA concentration and tumor volume and (b) between several individual fragment-based metrics, for cfDNA samples from patients with EwS.* Lines represent linear regression models fitted on the data; shaded areas represent the 95% confidence interval of the regression (estimated by bootstrapping). Bold font indicates significant correlations. p-values (two-sided; without corrections for multiple testing) were calculated using SciPy's stats.pearsonr and stats.kendalltau functions.

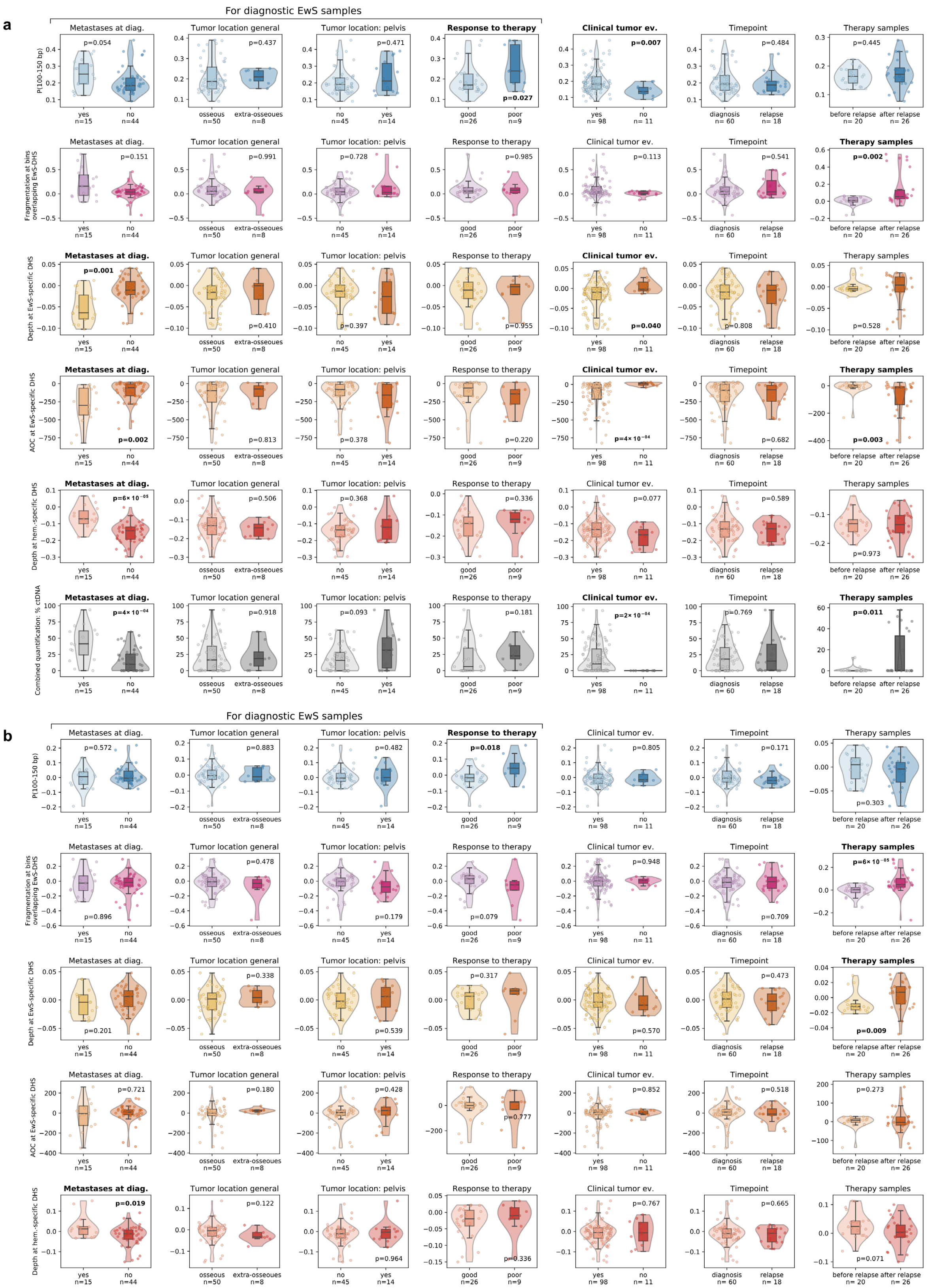

Supplementary Figure 10. *Associations between fragment-based metrics and several binary clinical metrics for samples from patients with EwS*, (a) before and (b) after correction for the following factors: genetic-based ctDNA quantification, input for library (ng), PCR cycle number, age at sample collection, and sex. p-values are based on Mann-Whitney U tests (two-sided; not corrected for multiple testing). Metastases status, tumor location, and response to therapy were evaluated for diagnostic samples only. Boxes correspond to interquartile ranges (IQR), black lines indicate the median, and the whiskers extend to the lowest or highest data points that are still within 1.5 IQR of the bottom or top quartile, respectively.

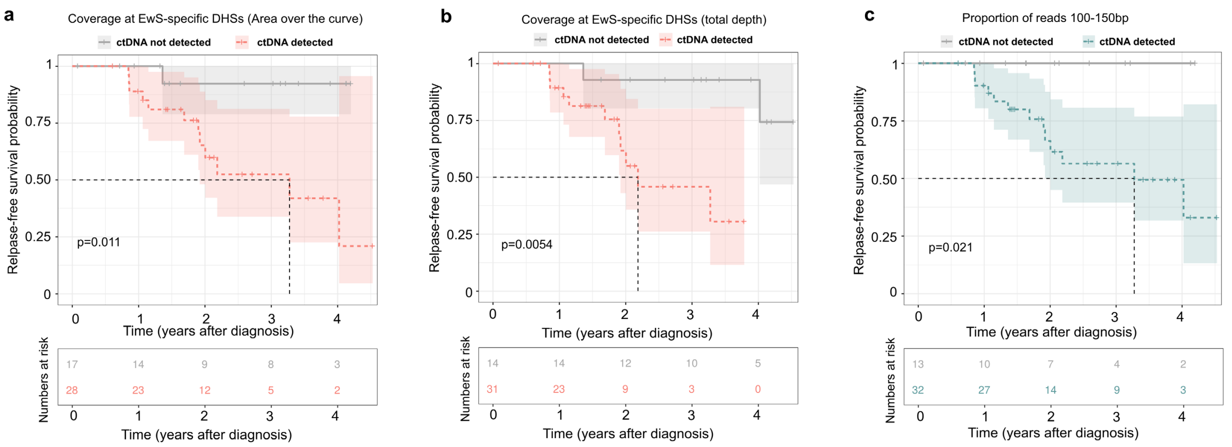

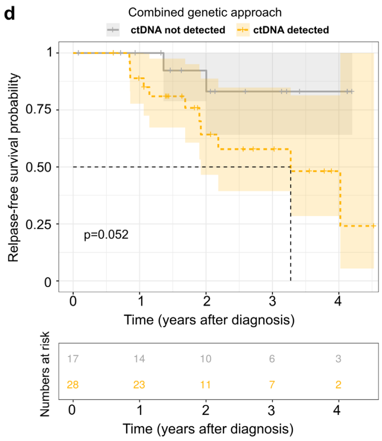

e

Metric for relapse free survival since diagnosis	p-value in multivariate coxPH model with sex and age	Hazard ratio (95% confidence interval)
Coverage at EwS-specific DHSs (total depth)	0.0421	8.75 (1.08-70.9)
Coverage at EwS-specific DHSs (AOC)	0.0593	0.0593 (0.93-57.0)
Proportion of fragments 100-150 bp	N/A	N/A
Combined genetic apporach	0.164	3.00 (0.64-14.2)

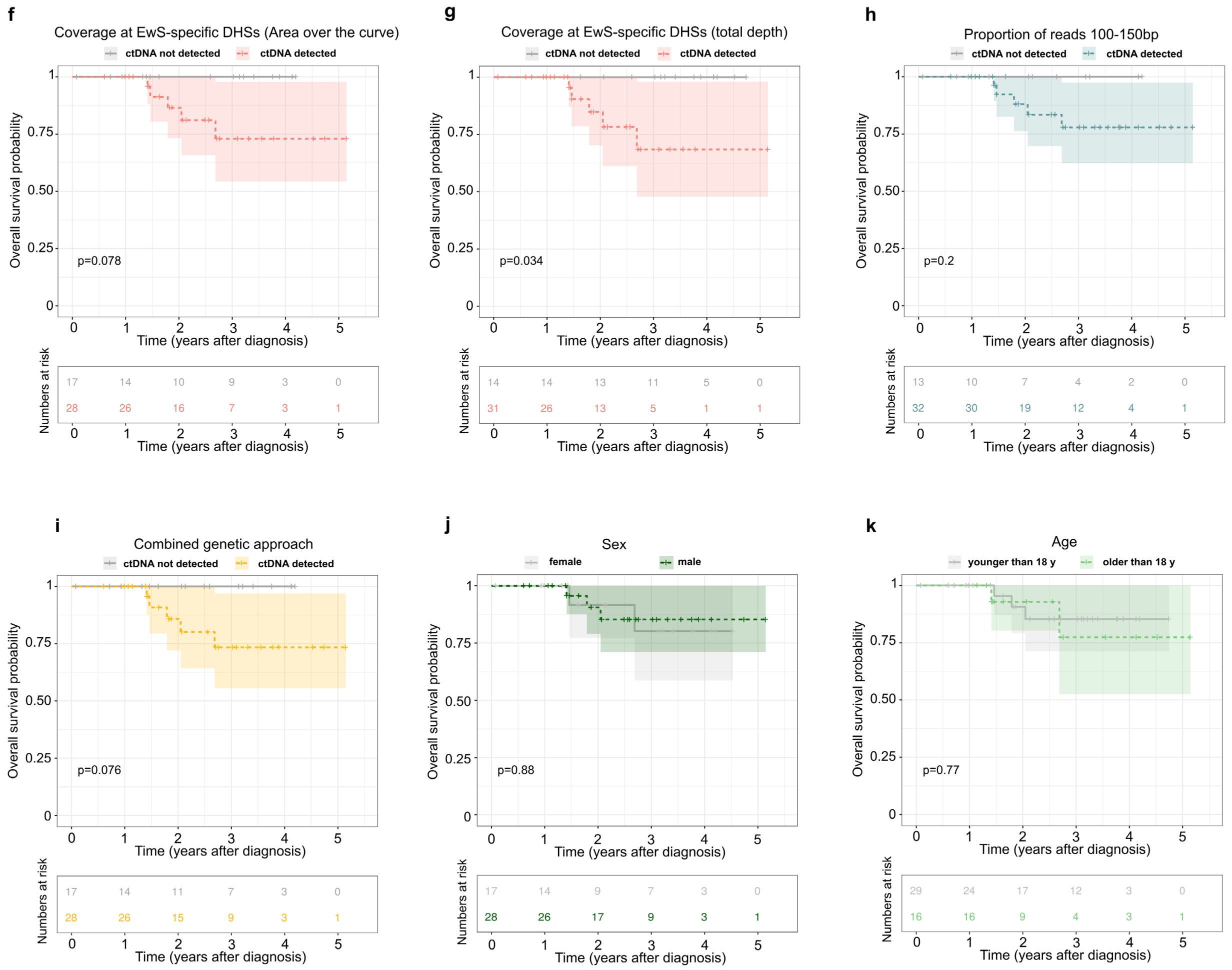

Supplementary Figure 11. *Kaplan-Meier curves showing differences in overall (OS) and relapse-free survival (RFS, measured from time of diagnosis) between patients with localized EwS, based on the detection of ctDNA at diagnosis.* For fragment-based approaches, |zscore vs healthy controls| > 2 was used as a detection threshold. Panels (a-e) show associations with RFS; panels (f-k) show associations with OS. The following metrics were evaluated: AOC and depth of the coverage drop at EwS-specific DHSs (a-b, f-g); the proportion of fragments in the range 100-150bp (c and h); and genetic tumor evidence based on the combination of ddPCR / WGS for breakpoint detection and CNA detection using ichorCNA (d and i). Panel (e) shows the results of multivariate cox-proportional hazards models that additionally included age and sex as covariates (as implemented in the coxph function of the survival R package; p-values are based on two-sided Wald tests). The proportional hazards assumption was verified in all cases using the cox.zph function. Note that for the associations of RFS with global fragment size, the application of the CoxPH model was not possible as none of the patients without detected ctDNA died in the observed period. As this applied also to all associations with OS, lack of association of OS with sex (j) and age discretized to below or above 18 years old (k) are also shown. The p-values shown in panels a-d and f-k were calculated using two-sided log-rank tests.

Supplementary References

1. Adalsteinsson, V.A.*, et al.* Scalable whole-exome sequencing of cell-free DNA reveals high concordance with metastatic tumors. *Nature communications* **8**, 1324 (2017).
2. Benjamini, Y. & Speed, T.P. Summarizing and correcting the GC content bias in high-throughput sequencing. *Nucleic acids research* **40**, e72 (2012).
3. Cristiano, S.*, et al.* Genome-wide cell-free DNA fragmentation in patients with cancer. *Nature* **570**, 385-389 (2019).
4. Ulz, P.*, et al.* Inference of transcription factor binding from cell-free DNA enables tumor subtype prediction and early detection. *Nat Commun* **10**, 4666 (2019).
