# Supplementary material for: Multimodal analysis of cell-free DNA whole-genome sequencing for pediatric cancers with low mutational burden
Source: Nat Commun. 2021 May 28;12:3230. doi: 10.1038/s41467-021-23445-w (PMC8163828; doi:10.1038/s41467-021-23445-w)
Supplement: Supplementary file 2 — Description of Additional Supplementary Files [file 41467_2021_23445_MOESM2_ESM.pdf]

Description of Additional Supplementary Files

File Name: Supplementary Data 1.

Description: Sample annotations and clinical data for the analyzed cfDNA samples from patients with EwS, patients with other pediatric sarcomas, and healthy individuals (sheet 1); sex and age statistics of the study cohort (sheet 2); molecular characteristics of the cfDNA samples and library preparation details (sheet 3); summary statistics of the whole genome sequencing data (sheet 4); clinical blood test results at the time of cfDNA sample collection (sheet 5).

File Name: Supplementary Data 2.

Description: Quantification of tumor-derived cfDNA based on EWS-Ets fusion breakpoint detection from whole genome sequencing data, CNAs inferred with ichorCNA, EWS-Ets fusion detection by ddPCR, and a handcurated consensus quantification combining the results of all three genetic methods (sheet 1); fusion breakpoints for each cfDNA sample from whole genome sequencing data (sheet 2); ddPCR primers and probes used in this study (sheet 3); quantification of tumor-derived cfDNA based on CNAs before and after filtering for short fragments / in silico size selection (sheet 4).

File Name: Supplementary Data 3.

Description: Quantitative metrics of cfDNA fragment size for individual cfDNA samples, calculated from the global fragment size distribution of all analyzed cfDNA samples

File Name: Supplementary Data 4.

Description: Overview of the low-coverage whole genome sequencing (lcWGS) and reduced representation bisulfite sequencing (RRBS) data for matched tumor samples, providing a list of the included samples (sheet 1); sequencing statistics for lcWGS (sheet 2) and RRBS (sheet 3); hand-curated comparison of CNAs in cfDNA and matched tumor samples (sheet 4).

File Name: Supplementary Data 5.

Description: Regional fragmentation profiles (z-scored log2 of the S/L ratios) and CNA status per chromosome arm for each cfDNA sample (sheet 1); p-values per chromosome arm for differences between EwS samples with genetic evidence (but without detected CNAs on that arm) and other groups of samples (sheet 2).

File Name: Supplementary Data 6.

Description: Detailed results of the LOLA-based region-set enrichment analyses (Fig. 4d), showing enriched region sets for: CNA-neutral bins with shorter (sheet 1) or longer (sheet 2) fragments in EwS samples with detectable tumor-derived cfDNA (based on genetic evidence); CNA-neutral bins with shorter (sheet 3) or longer (sheet 4) fragments in EwS samples without detectable tumor-derived cfDNA; CNA-neutral bins with shorter (sheet 5) or longer (sheet 6) fragments in non-EwS pediatric sarcoma samples with detectable tumor-derived cfDNA; and randomly selected bins in EwS samples with detectable tumor-derived cfDNA (sheet 7).

File Name: Supplementary Data 7.

Description: Changes in the regional fragmentation profiles (z-scored log2 of the S/L ratios) over time for seven patients with cfDNA samples and genetic evidence of tumor-derived cfDNA both at diagnosis and relapse (Supplementary Fig. 3c). Bins are ranked by their mean absolute change across patients.

File Name: Supplementary Data 8.

Description: Region sets used by LIQUORICE for regions-of-interest based analysis of cfDNA fragmentation: EwS-specific DNase I hypersensitive sites (DHSs) (sheet 1), EWS-FLI1 binding sites (sheet 2), EWSFLI1 correlated enhancers (sheet 3), EWS-FLI1 anti-correlated enhancers (sheet 4), hematopoietic-specific DHSs (sheet 5), liver-specific DHSs (sheet 6), universal DHSs (sheet 7), and alveolar rhabdomyosarcoma (ARMS)-specific DHSs (sheet 8).

File Name: Supplementary Data 9.

Description: Quantification of coverage signal in each cfDNA sample using multiple metrics, for the following regions-of-interest: EwS-specific DHSs (sheet 1), EWS-FLI1 binding sites (sheet 2), EWS-FLI1 correlated enhancers (sheet 3), EWS-FLI1 anti-correlated enhancers (sheet 4), hematopoietic-specific DHSs (sheet 5), liverspecific DHSs (sheet 6), universal DHSs (sheet 7), and alveolar rhabdomyosarcoma (ARMS)-specific DHSs (sheet 8).

File Name: Supplementary Data 10.

Description: Results of the machine-learning-based detection and classification of EwS cfDNA samples. Prediction summaries for the distinction of EwS samples with clinical tumor evidence from healthy control samples based on the meta-learner (sheet 1); prediction summaries for the distinction of EwS and non-EwS sarcoma samples with genetic tumor evidence based on the coverage signal at EwS-specific DHSs (sheet 2).
