# Supplementary material for: Multimodal analysis of cell-free DNA whole-genome sequencing for pediatric cancers with low mutational burden
Source: Nat Commun. 2021 May 28;12:3230. doi: 10.1038/s41467-021-23445-w (PMC8163828; doi:10.1038/s41467-021-23445-w)
Supplement: Supplementary file 13 — Reporting Summary [file 41467_2021_23445_MOESM13_ESM.pdf]

nature research

Corresponding author(s): Eleni M. Tomazou
Christoph Bock

Last updated by author(s): Apr 27, 2021

Reporting Summary

Nature Research wishes to improve the reproducibility of the work that we publish. This form provides structure for consistency and transparency in reporting. For further information on Nature Research policies, see Authors & Referees and the Editorial Policy Checklist .

Statistics

For all statistical analyses, confirm that the following items are present in the figure legend, table legend, main text, or Methods section.

n/a	Confirmed	
☐	☒	The exact sample size (*n*) for each experimental group/condition, given as a discrete number and unit of measurement
☒	☐	A statement on whether measurements were taken from distinct samples or whether the same sample was measured repeatedly
☐	☒	The statistical test(s) used AND whether they are one- or two-sided<br>*Only common tests should be described solely by name; describe more complex techniques in the Methods section.*
☒	☐	A description of all covariates tested
☐	☒	A description of any assumptions or corrections, such as tests of normality and adjustment for multiple comparisons
☐	☒	A full description of the statistical parameters including central tendency (e.g. means) or other basic estimates (e.g. regression coefficient) AND variation (e.g. standard deviation) or associated estimates of uncertainty (e.g. confidence intervals)
☐	☒	For null hypothesis testing, the test statistic (e.g. *F*, *t*, *r*) with confidence intervals, effect sizes, degrees of freedom and *P* value noted<br>*Give P values as exact values whenever suitable.*
☒	☐	For Bayesian analysis, information on the choice of priors and Markov chain Monte Carlo settings
☒	☐	For hierarchical and complex designs, identification of the appropriate level for tests and full reporting of outcomes
☐	☒	Estimates of effect sizes (e.g. Cohen's *d*, Pearson's *r*), indicating how they were calculated

Our web collection on statistics for biologists contains articles on many of the points above.

Software and code

Policy information about availability of computer code

Data collection

Whole genome sequencing was performed at the Biomedical Sequencing Facility of CeMM and MedUni Vienna, using Illumina HiSeq 3000/4000 instruments (2x101bp or 2x75bp flowcells) or a NovaSeq 6000 instrument (S4 2x100bp flowcells). Base calls provided by the Illumina Realtime Analysis software were converted into BAM files using Illumina2bam (https://github.com/wtsi-npg/illumina2bam) and demultiplexed using BamIndexDecoder from the same package. Initial quality control was performed using the FastQC software (http://www.bioinformatics.babraham.ac.uk/projects/fastqc/). Adapter trimming, initial quality control, and read-level filtering were performed with fastp using default settings. Quality-filtered reads were mapped to hg38 using the BWA-MEM software with default settings. Samblaster was used to mark duplicates, which were subsequently removed.

Data analysis

The analysis source code used in this manuscript is provided on the Supplementary Website (http://ews-liquid-biopsy.computational-epigenetics.org) and on Zenodo (http://doi.org/10.5281/zenodo.4719434).

The following software tools and packages were used (as described in the Methods section):

Illumina2bam v1.19 (demultiplexing)
FastQC v0.11.8 (qc)
gem-mappability 20130406-045632 (LIQUORICE)
fastp 0.20.0 (pre-processing)
Samblaster 0.1.24 (duplicate marking)
BWA 0.7.17-r1188 (alignment)
QuantaSoft software v1.7.4 (ddPCR analysis)
Trimmomatic 0.32 (RRBS analysis)
BSMAP 2.90 (RRBS analysis)
MIRA v1.8.0 (RRBS anaylsis)
IGV 2.4.14 (breakpoint analysis)

BLAT web interface (breakpoint analysis)
bwa 0.7.15-r1140 (breakpoint analysis)
samtools 1.3.1 (various tasks)
picard 2.8.1 (various tasks)
bedtools v2.27.1 (various tasks)
ichorCNA, git commit 1d54a1f (CNA analysis)
HMMCopy, v1.2.0 (CNA analysis)
deeptools suite v3.1.2 (various tasks)
LOLA v1.1 (region-set enrichment analysis)
pyBigWig python package v0.3.11 (LIQUORICE)
H2O python package v3.20.0.8 (machine learning)
scikit-learn python package v0.19.1 (machine learning)
lmfit python package v0.9.12 (LIQUORICE)
survival R package v3.1-12 (survival analysis)

For manuscripts utilizing custom algorithms or software that are central to the research but not yet described in published literature, software must be made available to editors/reviewers. We strongly encourage code deposition in a community repository (e.g. GitHub). See the Nature Research guidelines for submitting code & software for further information.

Data

Policy information about availability of data

All manuscripts must include a data availability statement. This statement should provide the following information, where applicable:

- Accession codes, unique identifiers, or web links for publicly available datasets
- A list of figures that have associated raw data
- A description of any restrictions on data availability

The sequence data has been deposited at the European Genome-phenome Archive (EGA), which is hosted by the EBI and the CRG, under accession number EGAS00001005127 (https://ega-archive.org/studies/EGAS00001005127). This data is available under a controlled access regimen to ensure the protection of personally identifiable data; access can be obtained by contacting E.M.T. Publically available sequencing data for cfDNA from healthy individuals were accessed via the EGA (EGAD00001005343, https://ega-archive.org/datasets/EGAD00001005343, and EGAD00001005339, https://ega-archive.org/datasets/EGAD00001005339). Pre-processed, de-identified data are available as an open-access online resource for viewing and download from the Supplementary Website (http://ews-liquid-biopsy.computational-epigenetics.org). The remaining data are available within the Article, Supplementary Information, or available from the authors upon request.

Field-specific reporting

Please select the one below that is the best fit for your research. If you are not sure, read the appropriate sections before making your selection.

- [x] Life sciences
- [ ] Behavioural & social sciences
- [ ] Ecological, evolutionary & environmental sciences

For a reference copy of the document with all sections, see nature.com/documents/nr-reporting-summary-flat.pdf

Life sciences study design

All studies must disclose on these points even when the disclosure is negative.

Sample size	This study included 200 plasma samples from 95 patients with EwS and 41 plasma samples from 31 patients with other types of sarcoma: EwS-like sarcoma (3 patients, two of which were positive for the CIC-DUX4 fusion gene), osteosarcoma (8 patients), rhabdomyosarcoma (12 patients), synovial sarcoma (3 patients), and other types of sarcoma (5 patients). Plasma samples from 22 healthy individuals (24-50 years old) were used as controls and were recruited for this study (7 individuals) or obtained via the Austrian Red Cross (15 individuals). The sample size was limited by sample availability for EwS, which is a a rare pediatric cancer. Validation in a large, prospective study cohort will be required to confirm the clinical associations and to qualify the method for routine clinical use.
Data exclusions	Samples that underwent in vitro size selection (N=15; French pilot cohort; Supplementary Table 2) were excluded from the analyses corresponding to Figures 2, 4, 6, and clinical associations, given that in vitro size selection has an impact on the fragment size distribution and therefore biases these analyses. Formaldehyde-fixed samples that showed signs of affected epigenetic properties (n=11, CCRI Biobank; Supplementary Data 2), were excluded from all epigenetics-based analyses (i.e., Figures 2, 4, 5, 6, and clinical associations). In order to ensure comparability of the classification performance of all metrics, these samples were completely excluded from the analysis corresponding to Figure 6. One sample with a very noisy CNA profile was excluded from Figure 2 (Supplementary Table 4). These data exclusion criteria were not pre-established, as an analysis of the fragment size distribution was not initially planned for the pilot study, and the impact of formaldehyde fixation only became apparent after sequencing of the affected samples.
Replication	We analyzed samples from 126 sarcoma patients and 22 internal plus 46 external healthy controls. Samples corresponding to the same patient are considered unique as they were collected at different time points during the disease course.
Randomization	No clinical trial and no treatment / control groups, therefore not applicable
Blinding	The study outcomes are quantitative and not subject to individual subjective judgement or interpretation; therefore blinding was not needed.

Reporting for specific materials, systems and methods

We require information from authors about some types of materials, experimental systems and methods used in many studies. Here, indicate whether each material, system or method listed is relevant to your study. If you are not sure if a list item applies to your research, read the appropriate section before selecting a response.

Materials & experimental systems

n/a	Involved in the study
☒	☐ Antibodies
☒	☐ Eukaryotic cell lines
☒	☐ Palaeontology
☒	☐ Animals and other organisms
☐	☒ Human research participants
☒	☐ Clinical data

Methods

n/a	Involved in the study
☒	☐ ChIP-seq
☒	☐ Flow cytometry
☒	☐ MRI-based neuroimaging

Human research participants

Policy information about studies involving human research participants

Population characteristics	This study included 200 plasma samples from 95 EwS patients and 41 plasma samples from 31 patients with other sarcomas: EwS-like sarcoma (3 patients, two of them having the CIC-DUX4 fusion gene); osteosarcoma (8 patients); rhabdomyosarcoma (12 patients); adult sarcoma (1 patient); synovial sarcoma (3 patients), and others (4 patients) (Supplementary Data 1). 49 patients were female, 75 patients were male. The median age at diagnosis was 14 years (min=0.8y, max=52y, Q1=9y, Q3=17y). Plasma samples from 22 healthy donors were used as controls. All healthy individuals were adults, mainly young adults (range 24-50 years, median=32y, 9 female and 13 male).
Recruitment	In total, we analyzed 263 plasma samples obtained from the following institutions: St. Anna Kinderspital, Vienna, Austria (55 samples); St. Anna CCRI biobank, Vienna, Austria (35 samples); Red Cross, Vienna, Austria (15 samples); Institute Curie, France (25 samples); University Hospital Erlangen, Germany (99 samples); and Oslo University Hospital, Norway (34 samples). Most of the patients with EwS included in this study were treated according to the EWING2008 protocol or slight variations of it. Patients from Norway were treated according to the ISG/SSG III protocol. Healthy controls were recruited for this study via personal communications (n=7), obtained via the Austrian Red Cross (n=15), or obtained from public datasets (n=46, EGAD00001005343, EGAD00001005339). For the healthy controls, we do not expect a substantial correlation between the cfDNA composition of blood plasma and the willingness to take part in this study. Therefore self-selection bias is not plausible here.
Ethics oversight	All samples were obtained with informed consent and with approval by the following review boards: Ethics Committee of the Medical University of Vienna (1292/2018), CPP SUD-EST IV, CPP 14/070, EE2012 study (reference number A 14-419), CPP ILE DE FRANCE III, CPP 3272, MAPPYACT study (reference number 2015-A00464-45), CPP ILE DE FRANCE IV, CPP 56-14, NGSKids study (reference number 2014-A00701-46), Ethics Committee of the "Ärztekammer Westfalen-Lippe und der Westfälischen Wilhelms-Universität Münster" (2008–391-f-A; EudraCT 2008–003658-13 EWING2008), and Ethics Committee for Medical Research in South-eastern Norway (17866).

Note that full information on the approval of the study protocol must also be provided in the manuscript.
